# Activated Carbons from Hydrochars Prepared in Milk

**DOI:** 10.1038/s41598-019-53361-5

**Published:** 2019-11-18

**Authors:** Salwa Haj Yahia, Kian Keat Lee, Brahim Ayed, Niklas Hedin, Tamara L. Church

**Affiliations:** 1 Materials and Environmental Chemistry, Stockholm University, Svante Arrhenius väg 16C, Stockholm, SE-106 91 Sweden; 2 Department of Chemical Engineering Process, National Engineering School of Gabes, University of Gabes, Gabès, Tunisia; 3 Laboratory of Materials, Crystal Chemistry and Applied Thermodynamics, Faculty of Science of Monastir, Monastir, Tunisia

**Keywords:** Chemistry, Materials chemistry

## Abstract

Hydrothermal carbonization converts organics in aqueous suspension to a mixture of liquid components and carbon-rich solids (hydrochars), which in turn can be processed into activated carbons. We investigated whether milk could be used as a medium for hydrothermal carbonization, and found that hydrochars prepared from milk, with or without an added fibrous biomass, contained more carbon (particularly aliphatic carbon), less oxygen, and more mineral components than those prepared from fibrous biomass in water. Activated carbons produced from hydrochars generated in milk had lower specific surface areas and CO_2_ capacities than those from hydrochars formed in water; however, these differences disappeared upon normalizing to the combustible mass of the solid. Thus, in the context of N_2_ and CO_2_ uptake on activated carbons, the primary effect of using milk rather than water to form the hydrochar precursor was to contribute inorganic mass that adsorbed little CO_2_. Nevertheless, some of the activated carbons generated from hydrochars formed in milk had specific CO_2_ uptake capacities in the normal range for activated carbons prepared by activation in CO_2_ (here, up to 1.6 mmol g^−1^ CO_2_ at 15 kPa and 0 °C). Thus, hydrothermal carbonization could be used to convert waste milk to hydrochars and activated carbons.

## Introduction

Milk is produced on an enormous scale, and as a result, so is waste milk. In Europe, 13% of milk produced is wasted, and in North Africa and West and Central Asia, the value is 20%^1^. In both regions, 3.5% of the milk produced is wasted at the production phase^1^, where it could potentially be recovered relatively easily. Even in Sweden, where an exceptionally low percentage of milk is wasted at production, the amounts of milk waste generated are large. For example, 0.32% of milk produced at Swedish farms in 2011 was discarded at the farm, primarily following antibiotic treatment of the cows for mastitis; this amounts to more than 9000 tons of milk^2^. Related to the issue of waste milk is dairy wastewater, which is composed of milk as well as additional water and detergents used for cleaning and sanitizing equipment^3^.

Milk is an aqueous dispersion (in the case of homogenized milk) or suspension (non-homogenized milk) of fats, proteins, and sugars, and also contains inorganic cations including K^+^, Na^+^, Ca^2+^, and Mg^2+^^4^. Aqueous preparations of organics, including suspensions of biomass, can be converted to carbon-rich solids called hydrochars via hydrothermal carbonization, i.e. by heating to (typically) 180−250 °C under autogeneous pressure^5–10^. The ability to convert wet biomass is the main process-related advantage of hydrothermal over pyrolytic carbonization^7^. Yoghurt (10 wt% in water) has been converted to a hydrochar that was evaluated as fuel^11^. Additionally, the hydrothermal carbonization of milk has been used as the first step in the synthesis of antibacterial carbon dot−Ag nanoparticle composites^12^.

Hydrochars from biomass can be activated to give activated carbons^5,8,9,13–15^. Biomass types that have been converted to activated carbons using this method include many lignocellulosic waste products^16,17^, for example bark^14^, sawdust^15^, rye straw^18^, grasses^19,20^, horse manure^19^, beer waste^19^, japonica^20^, and sewage sludge^21^. However, hydrochars have also been prepared from biomass sources with higher fat content, in particular microalgae^22^, and subsequently processed into activated carbons. Activated carbons, including those derived from hydrochars, can be used as CO_2_ sorbents^7,23–25^, and small amounts of Ca^2+^ increased the CO_2_-uptake capacity of polymer-derived activated carbons^26^.

**Figure 1:**
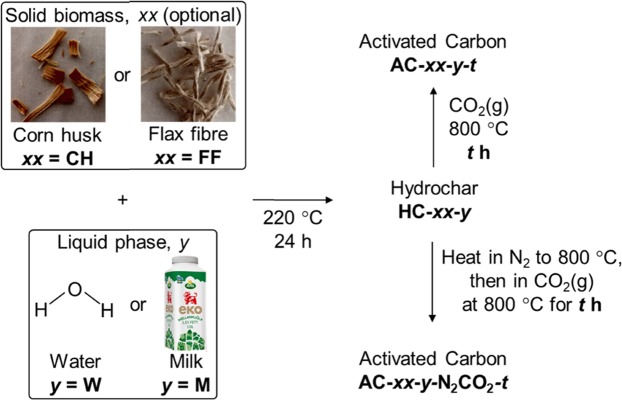
Summary of the synthesis of hydrochars (HCs) and activated carbons (ACs) described in this work, as well as the sample naming system (shown in bold characters).

To evaluate hydrothermal carbonization as a method of using waste milk, we converted homogenized milk to hydrochars that were characterized and activated to give activated carbons (Fig. 1) whose properties and CO_2_ sorption abilities were measured. Milk was also studied as a medium for the hydrothermal carbonization of fibrous biomass; thus, corn husk or flax fiber (corn husk is a waste product, and flax fibers are relevant to Swedish agriculture) were converted to hydrochars in water and in milk, and then activated to give activated carbons whose properties were compared.

## Experimental Section

The syntheses of hydrochars and activated carbons are described here; complete experimental and analytical details are given in the Supplementary Information.

### Hydrothermal carbonization

Flax fibers (unbleached, Växbo Lin, Sweden) and corn husks (removed from corn obtained from a local market) were divided into 1-cm pieces and allowed to dry at room temperature for 3 d to reach constant mass before hydrothermal carbonization. In each hydrothermal carbonization, a Teflon vessel was charged with liquid (80 or 200 mL of deionized water or milk), and solid biomass (0.1 g flax fiber or corn husks per mL water or milk) was added when desired. The vessel was sealed in an autoclave reactor, which was transferred to a Thermo Scientific Heraeus oven and heated at 200 °C h^−1^ to 220 °C, held at that temperature for 24 h, and then allowed to cool to room temperature at 80 °C h^–1^. The resulting solid was recovered by filtration, washed several times with deionized water, and dried at 100 °C for 24 h before it was crushed and sieved to give particles with d < 1 mm. This solid is labeled HC-*xx*-*y*, where *xx* = CH or FF for samples produced from corn husk or flax fiber, and *y* = W or M for samples produced in deionized water or milk. The hydrochar produced from milk without added solid is labeled HC-M.

### Activation

HC-*xx*-*y* (1–3 g) was charged into a vertical fixed-bed reactor and heated at 600 °C h^–1^ under 98 L h^–1^ gas (CO_2_ or N_2_) to 800 °C. The reactor was then held under 98 L h^–1^ CO_2_ flow for 4–20 h before it was allowed to cool to room temperature. The solid was removed from the reactor, crushed, and sieved to particles with d < 1 mm. The resulting samples that were both heated to and held at 800 °C under CO_2_ are labeled AC-*xx*-*y*-*t*, where *xx* and *y* give the details of hydrothermal carbonization (see above) and *t* gives the activation time in h. Samples that were heated to 800 °C under N_2_ and then held at that temperature under CO_2_ are labeled AC-*xx*-*y*-N_2_CO_2_-*t*.

## Results and Discussion

### Hydrochars

The hydrothermal carbonization of flax fiber and corn husk in water at 220 °C for 24 h gave hydrochars in 40 and 34% yield, in line with yields obtained in other studies of hydrothermal carbonization at moderate temperature and extended times^6^. More hydrochar was obtained when homogenized milk was used as the liquid for hydrothermal carbonization. The solid yields from hydrothermal carbonization in milk can be estimated by taking the combined mass of added solid plus solid components in the milk as the solid input; using this method, the yields from the hydrothermal carbonization of flax fiber and corn husk in milk were 81% and 70%.

**Figure 2:**
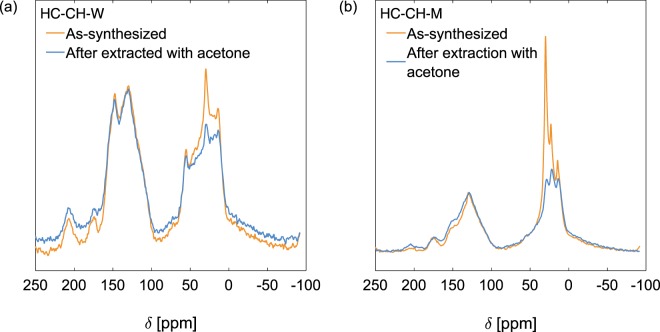
Solid-state {^1^H}^13^C NMR crosspolarization spectra of hydrochars (HC) produced in water or milk, as-synthesized and following extraction in refluxing acetone for 24 h. (**a**) HC-CH-W, and (**b**) HC-CH-M. Magic angle spinning at 14 kHz was used.

The hydrochars produced in milk contained more H and N, but less O, than their counterparts produced in water (Table 1). The greater H content was reflected in the IR spectra of the HC-*xx*-M (Supplementary Fig. S1a), which showed much more intense ν(C−H) bands, primarily associated with aliphatic C−H bonds (3000−2800 cm^−1^), than the spectra of the HC-*xx*-W. The ^13^C NMR spectrum of HC-CH-W (Fig. 2a) resembled that reported for HC produced from rye straw at 240 °C^27^, showing peaks for both saturated (δ < 80 ppm) and unsaturated (δ > 100 ppm) carbons, as well as unsaturated oxygenated groups such as carboxylic acids (δ ~ 175 ppm) and ketones (δ ~ 205 ppm). A very small peak at δ ~ 72 ppm may have indicated the presence of unreacted sugars or cellulose^27,28^. Extraction of HC-CH-W in acetone lowered the ^13^C NMR intensity associated with saturated carbons, in particular for the peak at δ ~ 30 ppm, and the concomitant loss of carboxylic acid and ketone carbons suggested that levulinic acid was a component of the extractable material, which was obtained as a darkly colored solid. Free levulinic acid has been detected in hydrochars from glucose^29^. In agreement with the IR results, the ^13^C NMR spectrum of HC-CH-M (Fig. 2b) revealed it to contain a much larger fraction of saturated carbons than HC-CH-W. Further, the fraction of the carbons that were oxygenated (δ ~ 150 and ~ 50 ppm for unsaturated and saturated carbons) was lower in the hydrochar produced in milk, consistent with the lower n_O_/n_C_ ratio observed for the hydrochars generated in milk (Table 1)^29^. After extraction with acetone, the fraction of saturated carbons in HC-CH-M fell, and the extract itself was a dark viscous oil. Fatty acids are not readily converted to hydrochar, but do adsorb onto hydrochars formed from sugars^30^; they can then be extracted using ethers^30,31^ or ethanol^32^. Therefore, the extractable saturated carbons on HC-CH-M were likely largely from fatty acids. The HC-*xx*-M had lower surface areas than the HC-*xx*-W (Table 1), and this difference is attributed to extractable molecules adsorbed on and in the pores of the HC-*xx*-M.

**Table 1:** Properties of hydrochars (HC) generated from hydrothermal carbonization in water or milk.

HC-*xx*-*y*		Elemental composition [% by mass]			Molar ratio^*a*^ [−]		*S*_BET_^*b*^	Res. mass^*c*^
*xx*	*y*	m_C_	m_H_	m_N_	n_H_/n_C_	n_O_/n_C_	[m^2^ g^−1^]	[%]
CH	W	71.0	4.73	1.71	0.79	0.23	18	0.54
	M	69.8	6.10	4.99	1.0	0.16	4.3	4.0
—	M	62.8	6.97	5.91	1.3	0.20	4.5	7.3
FF	W	66.1	4.85	0.37	0.87	0.32	28	0.09
	M	73.0	6.33	4.46	1.0	0.13	5.4	3.2

^a^n_O_ is approximated as n_O_ = [100 – mass % remaining after combustion to 800 °C – (m_C_ + m_H_ + m_N_)]/16. ^b^*S*_BET_ = Brunauer–Emmett–Teller surface area, calculated over P/P_0_ = 0.05–0.25. ^c^Res. mass = mass % remaining after combustion to 800 °C.

The HC-*xx*-W were more thermally stable at lower temperature, losing less than 5% of their mass when heated in air over 100–250 °C, whereas the HC-*xx*-M lost 15–20% of their mass in the same temperature range (Supplementary Fig. S1b). Heating HC-*xx*-W to 800 °C in air left very little residue (<0.6 wt%), whereas the hydrochars produced in milk retained 3–8% of their mass (Table 1), indicating that some of the mineral elements from the milk were retained. The IR spectra (Supplementary Fig. S1a) of the hydrochars produced in milk showed two peaks, at approximately 600 and 560 cm^−1^, that were not observed for the hydrochars produced in water. The positions and relative intensity of these peaks are consistent with those for vibrations associated with the phosphate groups of apatite^33^, and they may therefore be related to an inorganic phosphate.

### Activated carbons

The as-synthesized hydrochars were heated at 800 °C in CO_2_ for 4–20 h to give activated carbons. Very high capacities for CO_2_ sorption have been observed for hydrochars after activation with KOH^15^ or K_2_CO_3_^13^; however, the use of solid etchants requires an additional washing step in the material preparation, and KOH in particular is corrosive^34^, and we therefore focused on activation with CO_2_. Generally, hydrochars were heated in CO_2_ and then held at 800 °C under CO_2_, but a modified procedure was also tested for a few samples. Here, the solid was heated to 800 °C under N_2_ before the gas stream was changed to CO_2_ and the sample held at 800 °C for 20 h. The resulting activated carbons are distinguished with the term ‘N_2_CO_2_’ in the sample name.

**Figure 3:**
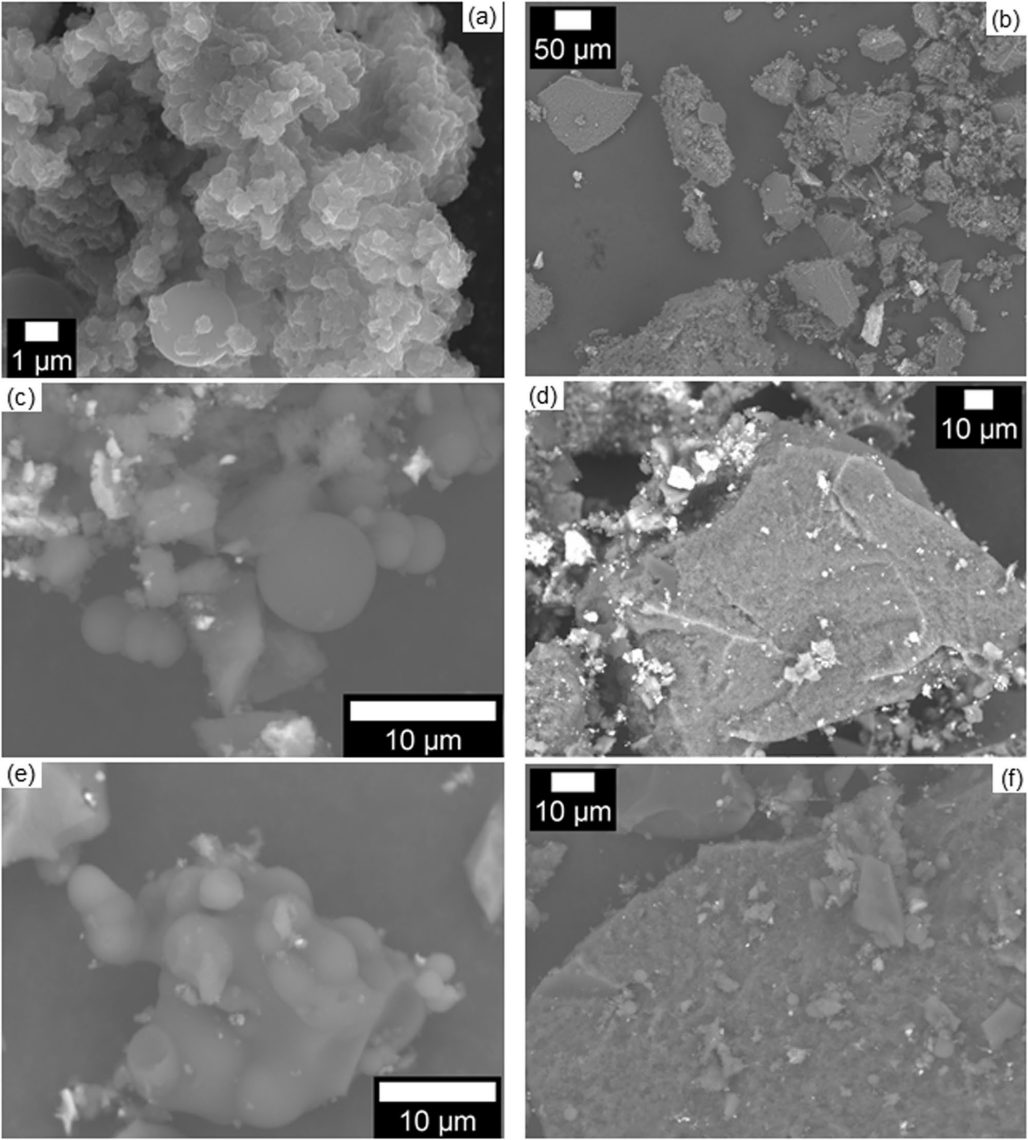
Scanning electron microscope images of activated carbons (ACs) generated from a hydrochar formed in milk without solid precursors. For each sample, a smaller particle is shown on the left, and a larger one on the right. (**a**,**b**) AC-M-4; (**c**,**d**) AC-M-10; (**e**,**f**) AC-M-N_2_CO_2_-20.

Scanning electron microscope images of activated carbons derived from the HC-M sample produced with no solid biomass (Fig. 3) showed two types of particles. The smaller particles (~5−50 μm; Fig. 3a,c,e) seemed smooth and were agglomerations of spheres that were reminiscent of the carbonaceous spheres seen in the hydrothermal carbonization of carbohydrates^27,35–38^, along with more irregular macroporous particles. Energy dispersive X-ray spectroscopy (EDS) of one such particle (Supplementary Fig. S2a) in AC-M-4 indicated that it was composed primarily of carbon and oxygen, but also contained small amounts of calcium, phosphorus, potassium, and magnesium. Larger (hundreds of μm), irregularly shaped particles with defined edges, sometimes bearing small spheres on their surfaces, were also present (Fig. 3b,d,f), and EDS showed one such particle to be composed of iron and oxygen, and to a lesser extent carbon (Supplementary Fig. S2b). We have previously observed Fe in activated carbons generated from hydrochars, even when no Fe precursor was added; this is derived from the stainless steel reactor used during the activation^39^.

The surface morphologies of flax fiber and corn husk (Supplementary Fig. S3a,b) were retained throughout hydrothermal carbonization and activation, with AC-FF-W-10 (Supplementary Fig. S3c) appearing as short fibers, and AC-CH-W-10 (Supplementary Fig. S3d) as broader sheets. These structures were also retained when milk was used in the hydrothermal carbonization (Supplementary Fig. S3e,f), but in that case were accompanied by the carbonaceous spheres and amorphous material seen in the activated carbons produced without solid biomass (Fig. 3).

**Figure 4:**
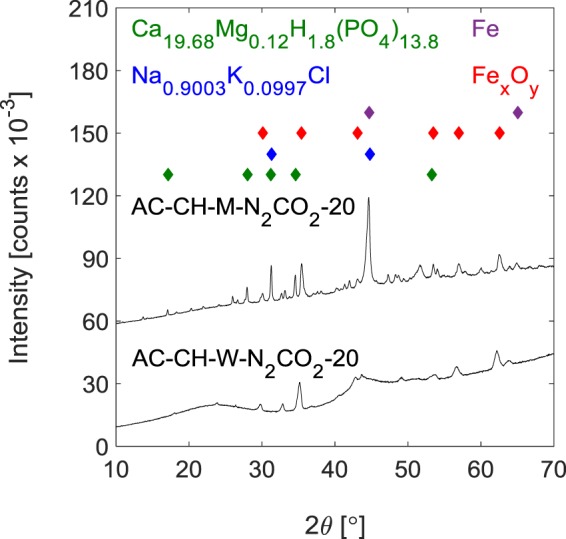
Powder X-ray diffraction patterns of selected activated carbons (ACs). Corn husk (CH) and liquid (water (W) or milk (M)) were heated in a sealed autoclave at 220 °C to form a hydrothermal carbon that was heated under N_2_ to 800 °C, then held at that temperature under CO_2_ for 20 h. Symbols above show the positions of the most intense peaks for relevant inorganic phases. The pattern for AC-CH-M-N_2_CO_2_-20 has been vertically offset for clarity. Diffraction patterns for all ACs are shown in Supplementary Fig. S4.

Activation increased the aromaticity of the carbonaceous solids, as n_H_/n_C_ fell in all cases (from 0.79–1.3 for hydrochars to 0.15–0.34 for activated carbons; cf. Tables 1 and 2). The formation of partially graphitized carbon was evinced by broad X-ray diffraction (XRD; Fig. 4 and Supplementary Fig. S4) peaks centered at 2θ = 23–25 and 43°, which correspond to the (002) and (10) planes of turbostratic carbon^40^, for most samples. These peaks were very weak for samples derived from milk without an additional biomass source (i.e. AC-M-*t*, Supplementary Fig. S4e), and for samples heated to 800 °C in N_2_ (Fig. 4, cf. Supplementary Fig. S4). In two samples that were examined with X-ray photoelectron spectroscopy (XPS of AC-CH-*y*-N_2_CO_2_-20 for *y* = W and M; see Supplementary Figs S5 and S6), the C 1s peaks included long slopes toward high binding energies, which supported the presence of graphitic or carbon black-type structures, though detailed deconvolution was not possible.

**Table 2:** Properties of the activated carbons prepared from the activation of hydrochars generated in water or milk.

AC-*xx-y-t*			Activated carbon (AC)									
*xx*	*y*	*t*	Yield [%]	Elemental composition [% by mass]			n_H_/n_C_ [−]	Non-combustible mass^a^ [%]	*S*_BET_^b^ [m^2^ g^–1^]	V_μ-pore_^c^ [cm^3^ g^–1^]	CO_2_ Uptake [mmol g^–1^]^d^ at 0 °C and *P*_CO2_=	
				C	H	N					15 kPa	101 kPa
CH	W	4	39	85.8	1.36	1.63	0.19	4.2	426	0.171	1.6	3.0
		10	50	88.8	1.35	1.72	0.18	6.2	413	0.186	1.6	3.1
		20	42	73.3	0.94	1.39	0.15	12	643	0.258	1.6	3.7
		N_2_CO_2_-20	41	75.3	1.20	1.49	0.19	11	748	0.299	1.7	3.9
	M	4	25	61.3	0.99	4.76	0.19	25	298	0.119	1.3	2.4
		10	26	65.0	1.26	3.88	0.23	30	457	0.181	1.6	3.1
		N_2_CO_2_-20	41	35.4	1.03	2.19	0.35	48	478	0.191	0.87	2.2
−^e^	M	4	16	43.6	1.16	3.51	0.32	28	260	0.103	1.0	2.0
		10	18	47.1	1.18	3.61	0.30	42	354	0.141	1.0	2.2
		N_2_CO_2_-20	18	53.4	1.03	4.41	0.23	28	446	0.179	1.1	2.5
FF	W	4	44	93.3	1.17	<0.10	0.15	1.5	502	0.200	1.7	3.4
		10	42	91.7	1.28	<0.10	0.17	2.7	584	0.231	1.8	3.8
		20	40	89.4	1.18	<0.10	0.16	1.5	649	0.256	1.8	4.2
		N_2_CO_2_-20	21	76.9	1.12	<0.10	0.17	1.2	527	0.209	1.8	3.6
	M	4	40	74.4	1.23	3.91	0.20	12	376	0.149	1.5	2.7
		10	37	71.2	1.29	3.87	0.22	16	414	0.164	1.6	3.1
		N_2_CO_2_-20	21	50.0	1.09	2.39	0.26	44	398	0.159	0.94	2.2

^a^Non-combustible mass = percentage of mass that remains after heating the sample to 800 °C in 25 mL min^−1^ air. ^*b*^ *S*_BET_ = Brunauer–Emmett–Teller surface area^54^, calculated from the N_2_ adsorption isotherms (Supplementary Fig. S7) over P/P_0_ = 0.01–0.1. ^*c*^ V_μ-pore_ = micropore volume, calculated from the N_2_ adsorption isotherms (Supplementary Fig. S7) using the Dubinin–Radushkevich equation^55,56^ fitted over P/P_0_ = 0.0001–0.05. ^*d*^ CO_2_ uptake at 101 kPa is measured (isotherms in Supplementary Figs S9–S13); CO_2_ uptake at 15 kPa is interpolated from a two-site Langmuir fit to the isotherm data (details in Supplementary Information section S1.4). ^e^ No solid biomass source was added.

Whereas activation increased the carbon content of hydrochars formed in water (from 66–71 wt% C for HC-*xx*-W to 73–94 wt% C for AC-*xx*-W-*t*; cf. Tables 1 and 2), it decreased carbon content for most of the HC formed in milk (from 62–73 wt% C for HC-*xx*-M and HC-M to 35–75 wt% C for AC-*xx*-M-*t* and AC-M-*t*), because the removal (gasification) of organic material during activation caused the mineral components from the milk to make up a larger fraction of the activated carbons. Thus, although the residual mass of the AC-*xx*-W-*t* samples after combustion to 800 °C was never greater than 12%, it ranged from 11–48% for the AC-*xx*-M-*t* samples, with high values being observed particularly for samples with long activation times (Table 2).

There are likely multiple reasons for the larger mineral content of the activated carbons produced from milk-derived hydrochars as compared with activated carbons prepared from hydrochars generated in water. First, there were minerals in the milk, and hence more mineral components were observed in the hydrochars prepared from milk than from water, as expected (see above). Further, the yield from activation of an HC-*xx*-W was generally higher than that from activation of HC-*xx*-M when the solid feedstock and activation conditions were the same (Table 2); that is, more mass was removed from HC-*xx*-M. Thus, either the HC-*xx*-M were more readily etched than the HC-*xx*-W, or the mineral components from milk catalyzed the decomposition of the hydrochars, or both. We cannot reject the former hypothesis, as HC-*xx*-W and HC-*xx*-M were chemically different (Table 1, Fig. 2 and Supplementary Fig. S1); however, the metal ions present in milk likely also affected the process. Ca^2+^ catalyzes the gasification of biochars in CO_2_^41^, and K^+^ salts including KOH^15,35,42,43^, K_2_CO_3_^13,44^, KHCO_3_^45,46^, and K_2_C_2_O_4_^47^ are used to activate hydrochars and form activated carbons. Na^+^ salts can also be used in the preparation of activated carbons from hydrochars^9^. Elemental analysis of AC-FF-W-10 revealed no detectable K and only 0.03 wt% Ca; whereas AC-CH-M-10 contained 0.34 wt% K and 2.7 wt% Ca. Similarly, XPS (Supplementary Table S1) showed that AC-CH-M-N_2_CO_2_-20 contained K and Ca, whereas AC-CH-W-N_2_CO_2_-20 did not. Thus, the Ca^2+^ and K^+^ in milk were at least partially retained throughout hydrothermal carbonization and activation, and likely contributed to pore development in the activated carbons produced from HC-*xx*-M and HC-M.

The primary crystalline calcium-containing phases (Fig. 4 and Supplementary Fig. S4) in the activated carbons prepared from HC-*xx*-M were calcium phosphate (ICSD 00-003-0713) or calcium-rich mixed calcium magnesium phosphates, such as Ca_19.68_Mg_0.12_H_1.8_(PO_4_)_13.8_ (ICSD 01-079-2186) and Ca_19_Mg_2_(PO_4_)_14_ (ICSD 01-082-9075). The latter two are difficult to distinguish by powder XRD; however, XPS of AC-CH-M-N_2_CO_2_-20 (Supplementary Table S1) showed no Mg, so the more Mg-rich Ca_19_Mg_2_(PO_4_)_14_ is less likely to be important. Some potassium may have been present as K_n_Na_1−n_Cl (n = 0.2, ICSD 01-076-3440; n = 0.0997, ICSD 01-075-0305), but the small amounts involved and the presence of other phases render this assignment uncertain. Neither the calcium (magnesium) phosphates nor K_n_Na_1−n_Cl were significant in the activated carbons prepared from HC-*xx*-W. Two significant non-carbon phases were observed for activated carbons derived from hydrochars formed in both water and in milk; these were α-Fe (ISCD 01-071-4648), which gave rise to sharp peaks at 2θ = 44 and 65°, and an iron oxide (cubic Fe_3_O_4_, inverse spinel Fe_3_O_4_, or γ-Fe_2_O_3_; these produce similar powder XRD patterns), consistent with the observation of an iron oxide by EDS (Supplementary Fig. S2). Nevertheless, based on XPS the total Fe in the activated carbons was small (Supplementary Table S1).

**Figure 5:**
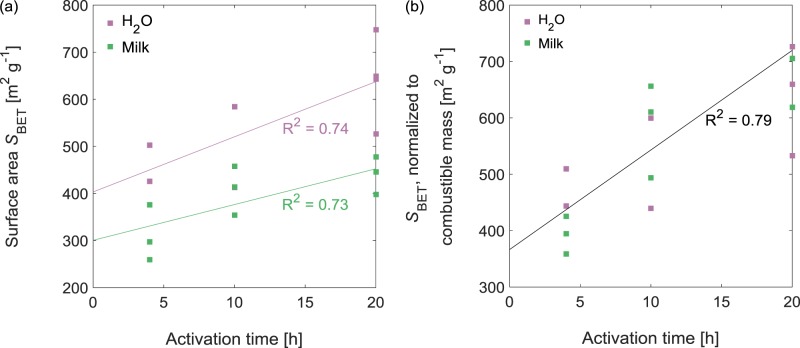
Brunauer–Emmett–Teller surface areas *S*_BET_ of the activated carbons derived from hydrochars produced in water or milk as functions of activation time. (**a**) Surface areas and (**b**) normalized surface areas. *S*_BET_ calculated from the N_2_ adsorption isotherms (Supplementary Fig. S7) over P/P_0_ = 0.01–0.1. Combustible mass is the mass fraction lost upon heating to 800 °C in 25 mL min^–1^ of dry air.

A consequence of the high inorganic content of the AC-*xx*-M-*t* was that they displayed lower apparent specific surface areas *S*_BET_ (300–480 m^2^/g) than the AC-*xx*-W-*t* (400–750 m^2^/g; Table 2, Fig. 5a; N_2_ sorption isotherms Supplementary Fig. S7). *S*_BET_ was correlated to activation time, but even the milk-derived activated carbons with the longest activation times had lower *S*_BET_ than most of the AC-*xx*-W-*t*. This difference did not reflect large discrepancies in the *S*_BET_ values of the carbonaceous portions of the activated carbons. Rather, when *S*_BET_ values were normalized to the combustible mass (fraction of mass lost upon heating to 800 °C in air) of each AC (Fig. 5b), there was no consistent difference between the activated carbons produced from HC-*xx*-W and HC-*xx*-M, though activation time remained a significant determinant of *S*_BET_. Thus, in terms of *S*_BET_, the primary impact of using milk as a starting material was to contribute low-surface-area inorganic mass.

**Figure 6:**
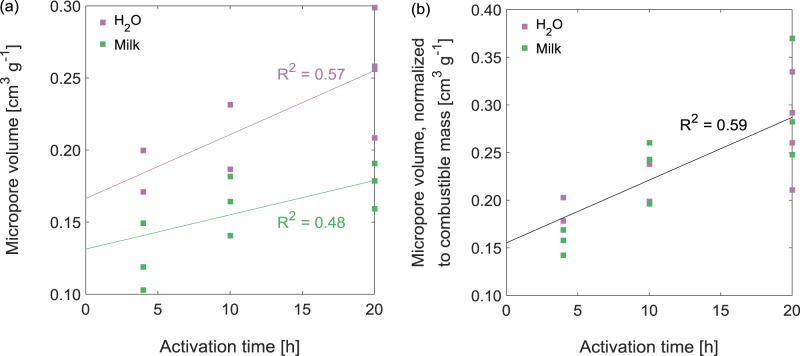
(**a**) Micropore volume V_μ-pore_ and (**b**) normalized micropore volume V_μ-pore_ for activated carbons (ACs) derived from hydrochars produced in water or milk as functions of activation time. Activation was under a flow of CO_2_ at 800 °C. Combustible mass is the fraction of mass lost when the AC is heated to 800 °C in 25 mL min^–1^ air.

AC-*xx*-M-*t* had different pore structures than AC-*xx*-W-*t* (Supplementary Figs S7 and S8). All of the activated carbons contained micropores, as indicated by N_2_ uptake at low pressure, but some also contained mesopores, as revealed by hysteresis in N_2_ uptake from P/P_0_ ~ 0.45. Activated carbons generated from HC-*xx*-M, even using shorter activation times, were mesoporous, especially when no solid precursor was used in the hydrochar (i.e. for the AC-M-*t* samples). This difference was likely due to the catalytic effect of the mineral components in HC-*xx*-M in etching the carbon; a larger average pore size has been observed in polymer-derived activated carbons when Ca^2+^ was added prior to activation^26^. Micropore volume (V_μ-pore_) increased with activation time, and the AC-*xx*-W-*t* samples consistently had higher V_μ-pore_ than the analogous AC-*xx*-M-*t* samples (Fig. 6a and Table 2). As was the case for the *S*_BET_, the difference in the V_μ-pore_ between AC-*xx*-W-*t* and AC-*xx*-M-*t* disappeared upon normalizing to the combustible mass of the AC (Fig. 6b).

As V_μ-pore_ is an excellent predictor of the CO_2_ sorption capacity of activated carbons, particularly under atmospheric CO_2_ pressure^48^, we expected the activated carbons generated from HC-*xx*-M to take up less CO_2_ than those from HC-*xx*-W. Indeed, the AC-*xx*-M-*t* generally took up less CO_2_ than the corresponding AC-*xx*-W-*t* (Supplementary Figs S9–S13, Table 2), both at 15 and 101 kPa, and CO_2_ uptake was correlated to V_μ-pore_, particularly at 101 kPa CO_2_ (Supplementary Fig. S14). Nevertheless, AC-CH-M-10 and AC-FF-M-10 each took up more than 1.6 mmol g^−1^ CO_2_ at 15 kPa and 0 °C, which is typical for activated carbons generated via activation in CO_2_ (Table 3). For example, it is in the range observed for activated carbons generated from the CO_2_-activation of other waste-derived hydrochars^19^, though lower than for activated carbons generated by CO_2_- or steam-activation of isolated^49^ or chemically modified^50^ cellulose.

**Table 3:** CO_2_ uptake capacity of some activated carbons derived from the activation of biomass or biomass-derived hydrochars (HCs) under CO_2_.

Precursor	Activation		CO_2_ uptake (0 °C, 15 kPa) [mmol g^−1^]	Ref.^a^
	*T* [°C]	*t* [h]		
Chitosan-crosslinked cellulose	900	1	2.29	^50^
HC-FF-W	800	10	1.8	This work
HC from RNA	800	16	2.0	^46^
HC from grass cuttings	800	2	1.8	^19^
Olive stones	800	6	1.8	^57^
HC-FF-M	800	10	1.6	This work
HC from biosludge	800	2	1.0	^19^

^a^Reference.

**Figure 7:**
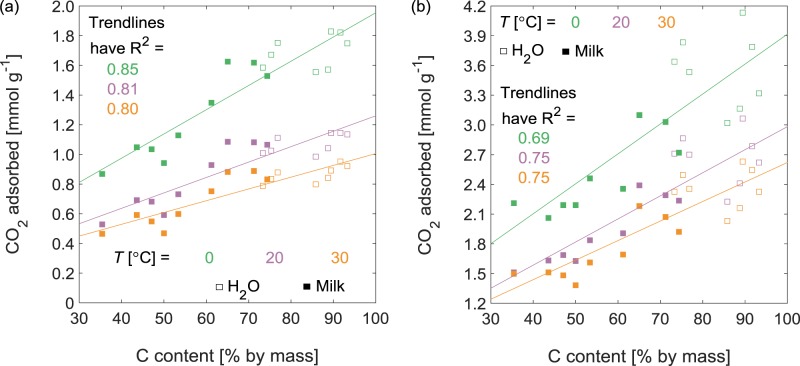
CO_2_ adsorption as a function of C content for activated carbons derived from hydrochars produced in water or milk. At *P*_CO2_ = (**a**) 15 kPa and (**b**) 101 kPa.

Overall, the best predictor of CO_2_ uptake capacity in the activated carbons produced here, from hydrochars generated in water or in milk, was their carbon content (Fig. 7). The correlation between C content and CO_2_ uptake was particularly strong at *P*_CO2_ = 15 kPa (Fig. 7a), the partial pressure of CO_2_ relevant to flue gas cleaning. Thus, despite that V_μ-pore_ increased with activation time (Fig. 6), the relationship between activation time and CO_2_ uptake was more complex (Supplementary Fig. S15), especially for activated carbons derived from hydrochars generated in milk. The HC-*xx*-M lost more C atoms (and thus had greater concentrations of inorganics) upon extended 20-h activation (Table 2), so the highest CO_2_ uptakes on AC-*xx*-M-*t* were obtained for activated carbons that had been activated for 10 h. Consistent with the dependence of CO_2_ capacity on the carbon content of the activated carbon, the CO_2_ uptake on AC-*xx*-W-*t* and AC-*xx*-M-*t* were not systematically different after normalizing to combustible mass. These values were clearly influenced by activation time though; longer times gave higher CO_2_ uptake per unit combustible mass at *P*_CO2_ = 101 kPa, but the opposite was true for CO_2_ uptake at *P*_CO2_ = 15 kPa (Supplementary Fig. S16). This difference can be understood in terms of pore development. CO_2_ uptake at low pressure depends on the volume of very small micropores (d ≤ 0.5 nm), whereas even larger micropores (d ≤ 1 nm) are important for CO_2_ uptake at *P*_CO2_ = 101 kPa^48^. Activation in CO_2_ for extended times produces more volume in larger micropores and less in smaller micropores^51^, and thus benefits CO_2_ uptake at *P*_CO2_ = 101 kPa.

The heats of adsorption *Q*_*st*_ for CO_2_ on the activated carbons produced from HC-*xx*-W and HC-*xx*-M (generally, *Q*_*st*_ = 22−32 kJ mol^−1^; Supplementary Fig. S17) were consistent with the values for the physisorption of CO_2_ on similar activated carbons. They were in the range observed on activated carbons derived from polymers pyrolyzed in the presence of KOH^52^ as well as on polymer-derived activated carbons containing CaO nanoparticles^26^, slightly higher than the values measured on a commercial NORIT activated carbons at similar loadings^53^, and slightly lower than those measured on an activated carbon obtained via the CO_2_-activation of a hydrochar formed from grass cuttings^19^.

Overall, the most important impact of using milk as the liquid phase in the hydrothermal carbonization to generate hydrochar-derived activated carbons for use as CO_2_ sorbents was to contribute inorganic mass that adsorbed little CO_2_. This inclusion of inorganic species had the net effect of producing activated carbons that took up less CO_2_ than analogous activated carbons made from hydrochars formed in water; however, the carbonaceous portions of the AC-*xx*-M-*t* and AC-*xx*-W-*t* took up similar amounts of CO_2_ in adsorption processes that were energetically similar. In this way, the AC-*xx*-M-*t* behaved, at least in the context of CO_2_ sorption, like composites of activated carbons and inorganics. Thus although the use of waste milk to produce hydrochar-derived activated carbons was clearly feasible, and some of these activated carbons had CO_2_ uptake capacities in the same range as other activated carbons produced using CO_2_ as the activation agent (1.6 mmol g^−1^ at 15 kPa and 0 °C), other uses of the AC-*xx*-M-*t* may be more interesting; future work will focus on applications that are favored by inorganic cations, such as calcium-catalyzed reactions.

## Supplementary information

Supplementary Information

## Data Availability

Figs 5 and 6 are constructed from data in Supplementary Figs S7 and S8, respectively, and Fig. 7 is constructed from data in Supplementary Figs S9−S13.
