# Supplementary material for: Activated Carbons from Hydrochars Prepared in Milk
Source: Sci Rep. 2019 Nov 18;9:16956. doi: 10.1038/s41598-019-53361-5 (PMC6861549; doi:10.1038/s41598-019-53361-5)
Supplement: Supplementary file 1 — Supplementary Information [file 41598_2019_53361_MOESM1_ESM.pdf]

Supporting information for:

# Activated Carbons from Hydrochars Prepared in Milk

*Salwa Haj Yahia,[†,‡] Kian Keat Lee,[†] Brahim Ayed,[‡,¶] Niklas Hedin,[†] Tamara L. Church[†,]**

*[†]Materials and Environmental Chemistry, Stockholm University, Svante Arrhenius väg 16C, Stockholm, SE-106 91, Sweden*

*[‡] Department of Chemical Engineering Process, National Engineering School of Gabes, University of Gabes, Tunisia*

*[¶] Laboratory of Materials, Crystal Chemistry and Applied Thermodynamics, Faculty of Science of Monastir, Tunisia*

** To whom correspondence should be addressed. tamara.church@mmk.su.se; +46 (8) 16 1260*

| **Item** | **Page** |
|---|---|
| S1. Experimental Details | S1 |
| S2. Additional characterization data for hydrochars | S4 |
| S3. Additional characterization data for activated carbons | S4 |
| S4. References | S16 |

## S1. Experimental Details

### *S1.1 Materials*

Corn husks were removed from corn purchased from a local market, and unbleached flax fiber was purchased from Växbo Lin (Sweden). Water was deionized. Milk was purchased from Arla, Sweden, and refrigerated prior to use. According to the producer, the milk contained 87% $H_2O$, 1.5% fat, 2.8% sugars, 3.9% protein, 0.1% NaCl, 0.12% $Ca^{2+}$. The measured pH of the milk was 6.83. $CO_2$ for activation was from Strandmøllen AB and 99.2% pure.

### *S1.2 Hydrothermal carbonization*

Flax fibers and corn husks were divided into 1-cm pieces and allowed to dry at room temperature for 3 d in order to reach constant mass before hydrothermal treatment. In each hydrothermal carbonization, a Teflon vessel was charged with liquid (80 or 200 mL of deionized water or milk), and solid biomass (0.1 g flax fiber or corn husks per mL water or milk) was added if desired. The vessel was sealed in an autoclave reactor, which was transferred to a Thermo Scientific Heraeus oven and heated at 200 °C $h^{-1}$ to 220 °C, held at that temperature for 24 h, and then allowed to cool to room temperature at 80 °C $h^{-1}$. The resulting solid was recovered by filtration, washed several times with deionized water, and dried at 100 °C for 24 h before it was crushed and sieved to give particles with d < 1 mm. This solid is labeled HC-*xx*-*y*, where *xx* = CH or FF for samples produced from corn husk or flax fiber, and *y* = W or M for samples produced in deionized water or milk. The char produced from milk without additional solid biomass is labeled HC-M.

*S1.3 Activation*

HC-*xx*-*y* (1–3 g) was charged into a fixed reactor bed and heated at 600 °C $h^{-1}$ under 98 L $h^{-1}$ gas ($CO_2$ or $N_2$) to 800 °C. The reactor was then held under 98 L $h^{-1}$ $CO_2$ flow for 4–20 h before it was allowed to cool to room temperature. The solid was removed from the reactor, crushed, and sieved to particles with d < 1 mm. The resulting samples that were both heated to and held at 800 °C under $CO_2$ are labeled AC-*xx*-*y*-*t*, where *xx* and *y* give the details of hydrothermal carbonization (see above) and *t* gives the activation time in h. Samples that were heated to 800 °C under $N_2$ and then held at that temperature under $CO_2$ are labeled analogously as AC-*xx*-*y*-$N_2CO_2$-*t*.

*S1.4 Characterization*

Surface textural properties of the activated carbons were investigated using a JEOL JSM-7000F microscope or a Hitachi TM3000 microscope, using an acceleration voltage 15 kV. Energy-dispersive X-ray spectroscopy (EDS) was obtained on the JEOL JSM-7000F at an acceleration voltage 15 kV. Powder X-ray diffraction (XRD) patterns were obtained using a Panalytical X'Pert PRO diffractometer using Cu $K\alpha_1$ radiation ($\lambda$ = 1.541 Å) and working at 45 kV and 40 mA. Diffractograms were collected at incident angles from $2\theta$ = 10 to 70° at a step size of 0.033° and each point was measured for 200 s. Elemental analyses (C, H, N, K, Ca, P) were performed by MEDAC Ltd. (Surrey, UK). X-ray photoelectron spectra were recorded at RI.SE (Sweden) on a Kratos AXIS UltraDLD spectrometer (Kratos Analytical, Manchester, UK under a vacuum ($P < 1.3 \times 10^{-10}$ bar) using a monochromatic Al X-ray. An electrostatic lens system was used due to magnetism in the sample, so the analysis area is ca 2–3 $mm^2$. Infrared (IR) spectra were measured on a Varian 610 IR spectrometer with a Specac single reflection attenuated total reflection (ATR) accessory and a deuterated triglycine sulfate (DTGS) detector, with the clean diamond element as the background. $\{^1H\}^{13}C$ NMR spectra were recorded under magic angle spinning (MAS) of 14 kHz at a frequency of 100.6 MHz. Ramped crosspolarization (1.5 ms) and SPINAL decoupling of the $^1H$ contributions during acquisition were used, and 16k transients were recorded for each spectrum. A Bruker Avance III 400 MHz spectrometer, a wide-bore magnet (9.4 T), and a 4-mm MAS probe head were used, and the $^{13}C$ chemical shift scale was calibrated externally using the methine signal of adamantane. Thermogravimetric analyses were measured on a TA Instruments Discovery TG. Samples were heated under 25 mL $min^{-1}$ instrument air at 5 °C $min^{-1}$ to 50 °C, held at that temperature for 30 min, and heated at 5 °C $min^{-1}$ to 1000 °C.

Gas Sorption

The nitrogen sorption isotherms of hydrochars were measured −196 °C on a Micromeritics Gemini VII sorption instrument. Samples were degassed under a flow of $N_2$ at 200 °C for 24 h prior to measurement. Brunauer–Emmett–Teller surface areas $S_{BET}$[1] were calculated over $P/P_0$ = 0.05–0.25. The sorption isotherms of activated carbon samples were measured on a Micromeritics ASAP 2020 instrument. Samples were degassed under vacuum at 50 °C for 12 h prior to measurement. $N_2$ isotherms were measured at −196 °C and $S_{BET}$ was calculated over $P/P_0$= 0.01–0.10. The total pore volume (in pores smaller than approximately 200 nm in diameter) is determined from the adsorption of $N_2$ at a $P/P_0$ value of 0.99. Pore size distributions were derived

from the adsorption branch of the isotherm for $P/P_0 < 0.35$ using the non-linear density functional theory (NL-DFT) functional developed by Micromeritics for the adsorption of $N_2$ on carbon with slit-like pores. Micropore volumes were calculated using the Dubinin−Radushkevich equation.[2,3] Generally, values calculated using the t-plot procedure were 10–20% smaller.

$CO_2$ sorption isotherms for each activated carbon were measured on the Micromeritics ASAP 2020 instrument directly after $N_2$ sorption and without further degassing. Isotherms were measured at 0, 20, and 30 °C. Initial plots of the sorption data as the ratio $P/q$ of gaseous and adsorbed $CO_2$ at equilibrium vs. the equilibrium pressure $P$ itself were not linear, indicating that a linearized Langmuir isotherm (Eq. 1)[4] was not sufficient to describe the adsorption of $CO_2$ on these materials. However, the data fit very well ($R^2 \geq 0.998$) to two-component Langmuir isotherms, which were calculated as follows. First, each isotherm was split into two pressure ranges, $P < 13$ kPa and $P > 13$ kPa, labelled A and B respectively. For each range, a linearized Langmuir isotherm was calculated according Eq. 1 to give approximate Langmuir coefficients $b'$ and maximum sorption amounts $q_S'$.

$$\frac{P}{q} = \frac{P}{q_s} + \frac{1}{q_s b} \tag{1}$$

For a given point at equilibrium, $P$ is the gas pressure in kPa above the surface and $q$ is the amount of gas adsorbed in $mmol_{CO2}/g_{sorbent}$. $q_S$ is the maximum sorption capacity in $mmol_{CO2}/g_{sorbent}$ of the solid at the applied temperature, and $b$ is the Langmuir constant in $kPa^{-1}$.

The values of $q_{S,A}'$, $b_A'$, $q_{S,B}'$, and $b_B'$ obtained from the Langmuir fits in pressure ranges A and B were used as initial values to fit the isotherms to the two-component non-linear Langmuir equation (Eq. 2). Non-linear least squares fitting was performed in Matlab using a Trust-Region algorithm, and the coefficients were restrained between 0 and ∞. Fitted curves are shown in the isotherms in Figures S6–10.

$$q = \frac{q_{s,A} b_A P}{1 + b_A P} + \frac{q_{s,B} b_B P}{1 + b_B P} \tag{2}$$

The values of $q_{S,A}$, $b_A$, $q_{S,B}$, and $b_B$ obtained by fitting the $CO_2$ adsorption isotherm for each sorbent and at each temperature were used to compute values of $P$ at various values of $q$, up to the maximum $q$ observed for that sorbent at 30 °C. The values of $P$ calculated for a given $q$ and sorbent were then compared according to Eq. 3 in order to derive the heat of adsorption $Q_{st}$ for $CO_2$ on that sorbent and at that degree of coverage. A linear relationship between ($lnP$) and ($T^{-1}$) was assumed in all cases, and correlation coefficients $R^2$ were almost always >0.995, although in one case ($CO_2$ sorption on AC-M-4 at $q = 0.05$), a low $R^2$ of 0.990 was obtained, along with an anomalously low $Q_{st}$ value of 22 kJ/mol. Thus the impact of temperature on sorption may have been complex in this instance.

$$Q_{st} = -\mathrm{R}\frac{\delta(lnP)}{\delta\left(\frac{1}{T}\right)} \tag{3}$$

R is the ideal gas constant.

## S2. Additional characterization data for hydrochars

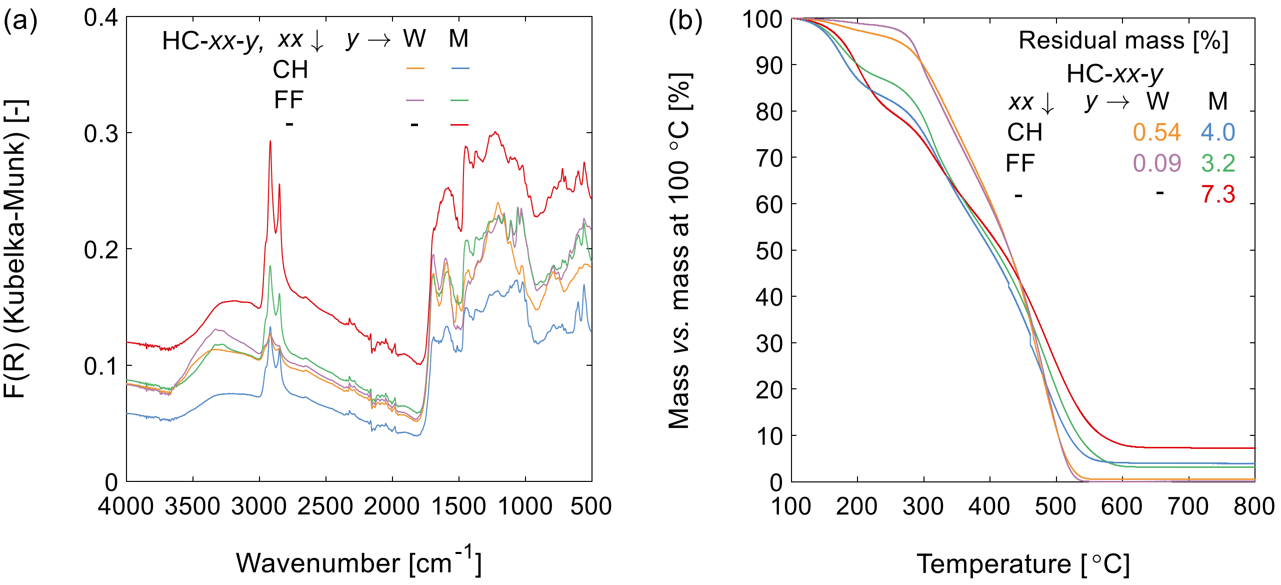


**Figure S1**. Characterization of hydrochars (HCs) produced by heating corn husk, flax fiber, or neither with water or milk in a sealed autoclave at 220 °C. (a) Attenuated total reflectance IR spectra and (b) thermogravimetric analyses of hydrochars. For thermogravimetric analysis, samples were heated under 25 mL min$^{-1}$ instrument air at 5 °C min$^{-1}$ to 50 °C, held at that temperature for 30 min, and then heated at 5 °C min$^{-1}$ to 800 °C.

## S3. Additional characterization data for activated carbons

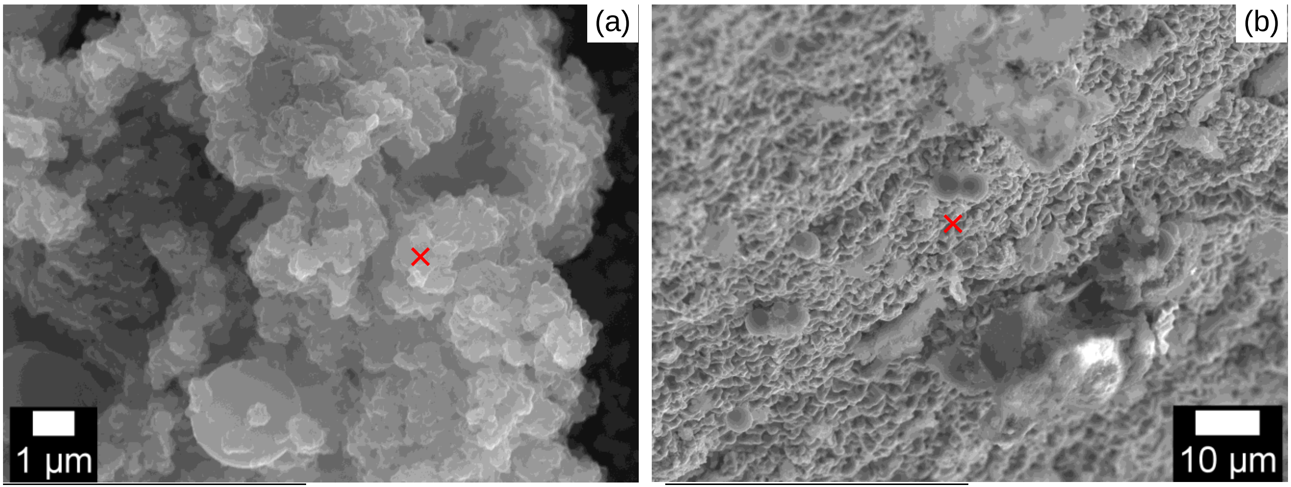


| Element | Content | |
|---|---|---|
| | [wt%] | [at%] |
| C[a] | 73.6 | 81.3 |
| O | 19.4 | 16.1 |
| Mg | 0.34 | 0.18 |
| P | 2.42 | 1.04 |
| K | 0.77 | 0.26 |
| Ca | 3.46 | 1.14 |

| Element | Content | |
|---|---|---|
| | [wt%] | [at%] |
| C[a] | 18.5 | 32.0 |
| O | 40.7 | 52.8 |
| Fe | 40.9 | 15.2 |

**Figure S2**. Energy dispersive X-ray spectroscopic investigations of two particles in a sample of AC-M-4. (a) Scanning electron microscope image of the smaller examined particle. (b) Scanning electron microscope image of the larger examined particle. An × marks the point on each particle where energy dispersive X-ray spectra were measured, and the resulting elemental compositions are displayed below each image. Note that the carbon tape holding the samples may contribute to the elemental compositions.

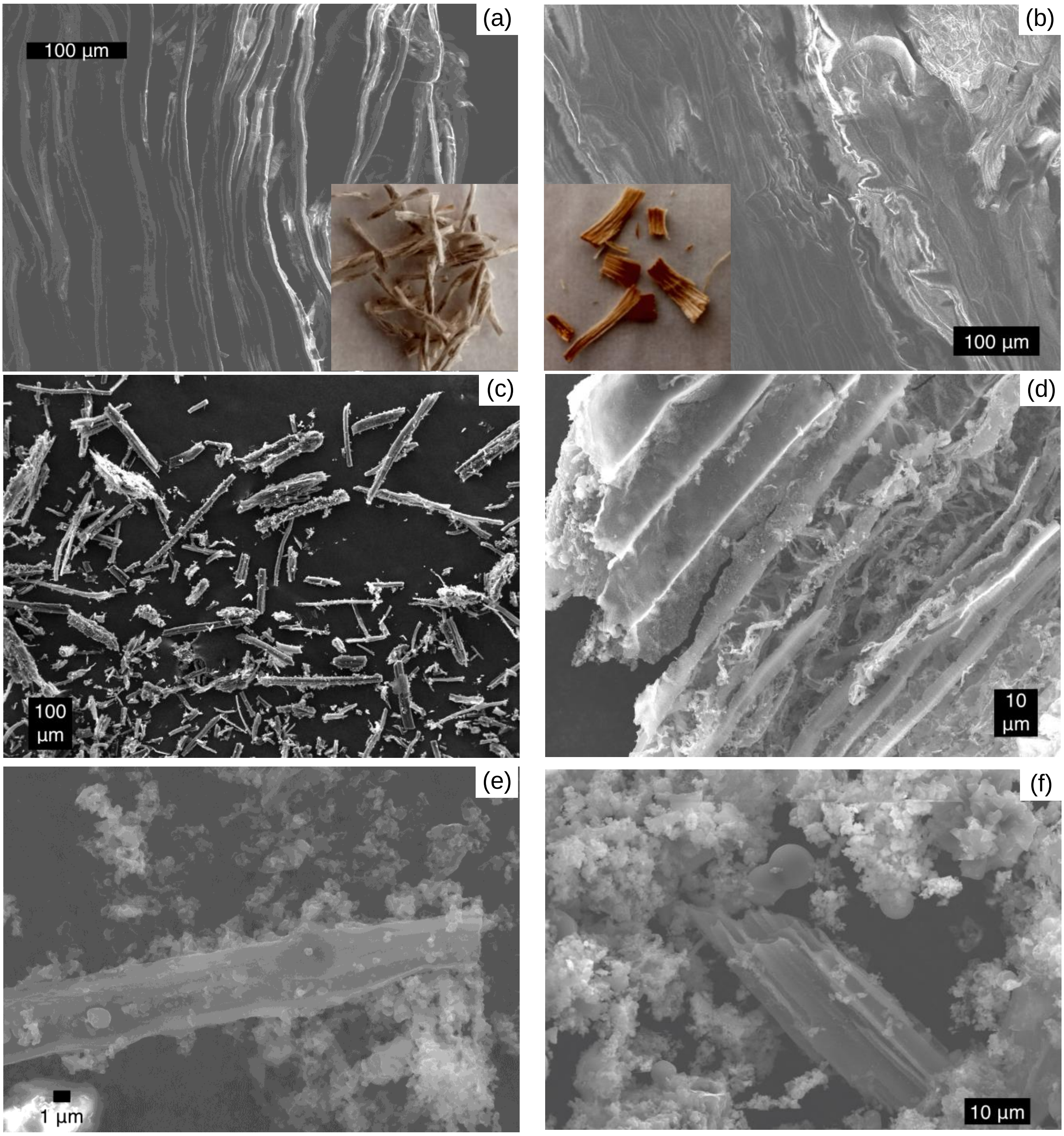


**Figure S3**. Scanning electron microscope images of activated carbons and their biomass precursors. (a) Flax fiber (inset: digital photo); (b) corn husk (inset: digital photo); (c) AC-FF-W-10; (d) AC-CH-W-10; (e) AC-FF-M-10; (f) AC-CH-M-10.

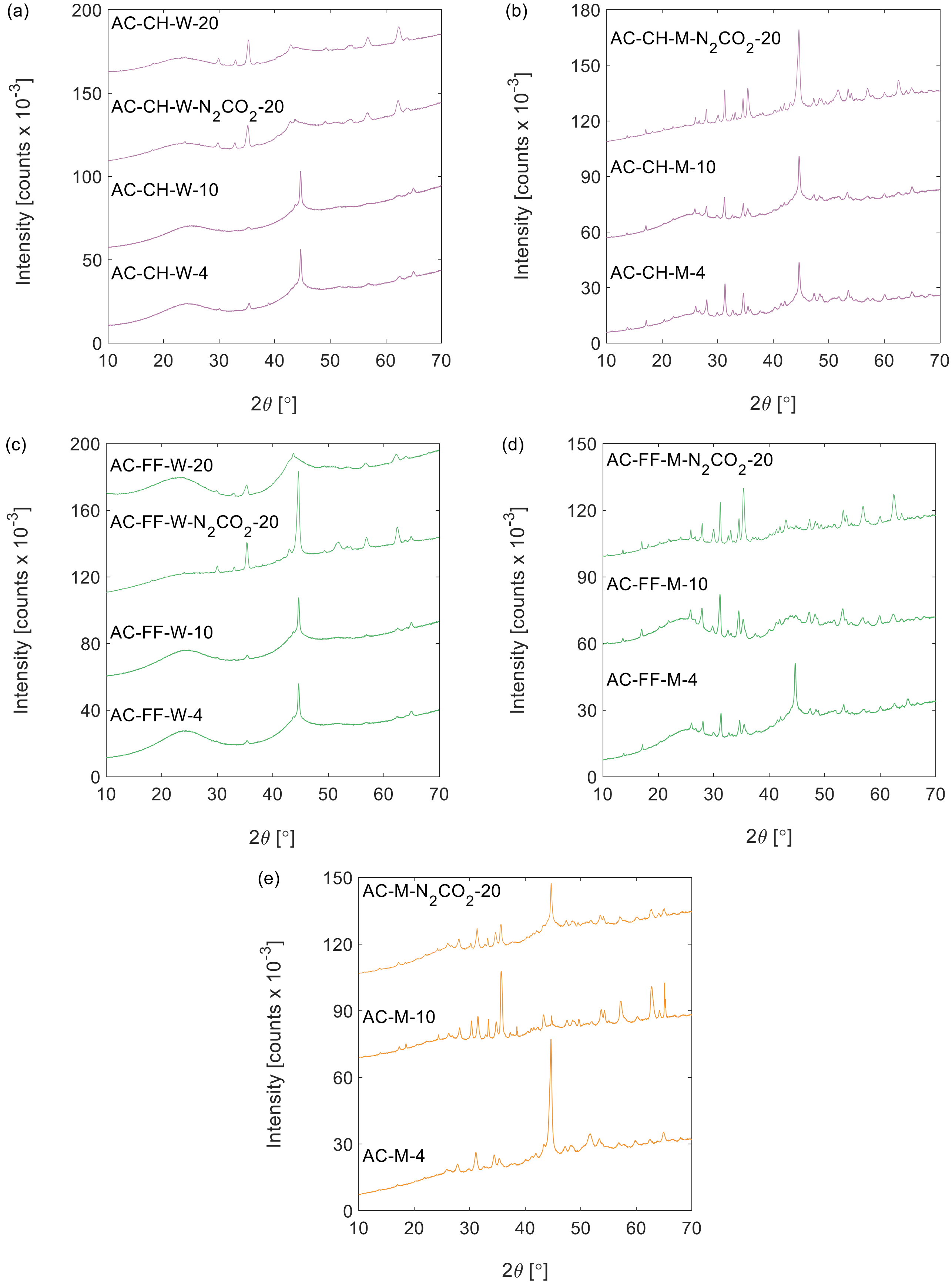


**Figure S4**. Powder X-ray diffraction patterns of different activated carbons (ACs). The starting solid (corn husk (CH), flax fiber (FF), or neither) and liquid (water (W) or milk (M)) were heated in a sealed autoclave at 220 °C to form a hydrochar (HC-*xx*-*y*) that was activated in $CO_2$ at 800 °C for the indicated time. Activated carbons produced from: (a) HC-CH-W; (b) HC-CH-M; (c) HC-FF-W; (d) HC-FF-M; (e) HC-M. Patterns have been vertically offset for clarity.

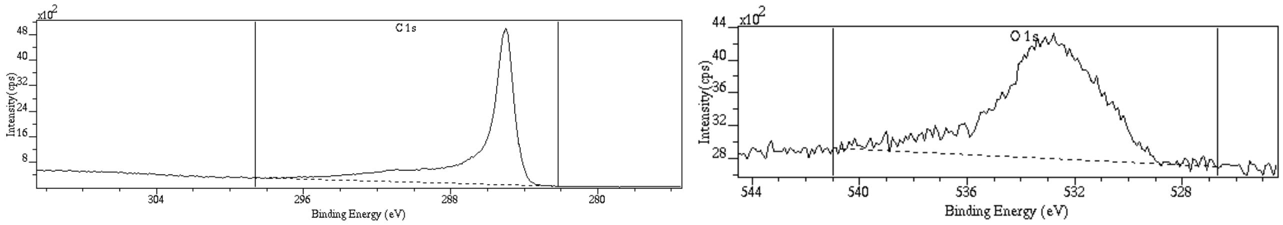


**Figure S5**. High-resolution C 1s and medium-resolution O 1s X-ray photoelectron spectra for AC-CH-W-$N_2CO_2$-20. cps = counts per second.

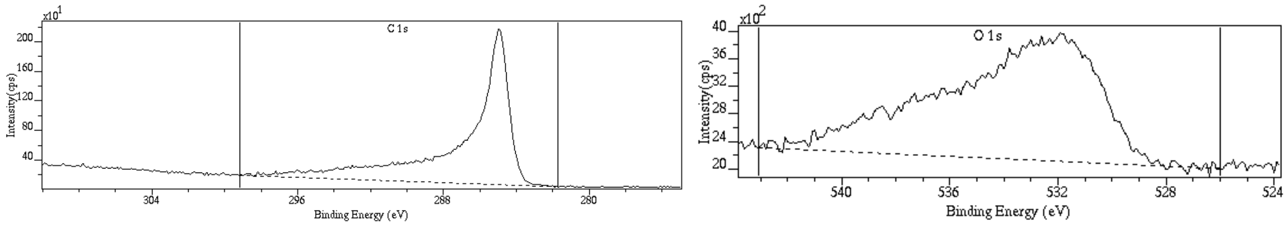


**Figure S6**. High-resolution C 1s and medium-resolution O 1s X-ray photoelectron spectra for AC-CH-M-$N_2CO_2$-20. cps = counts per second.

**Table S1**. X-ray photoelectron spectroscopic analysis of selected activated carbons (ACs) generated from hydrochars in water or milk.

| Sample | Elemental content [at%] | | | | | | | | | |
|---|---|---|---|---|---|---|---|---|---|---|
| | C | O | N | Fe | K | P | Ca | Mg | Na | Cl |
| AC-CH-W-$N_2CO_2$-20 | 95.2 | 3.6 | 1.1 | –[a] | –[a] | –[a] | –[a] | –[a] | 0.1[b] | –[a] |
| AC-CH-M-$N_2CO_2$-20 | 83.8 | 9.7 | 3.1 | 0.3[b] | 0.9 | 1.1 | 1.0 | –[a] | 0.1[b] | –[a] |

[a]Not detected. [b]Peak was near the level of noise.

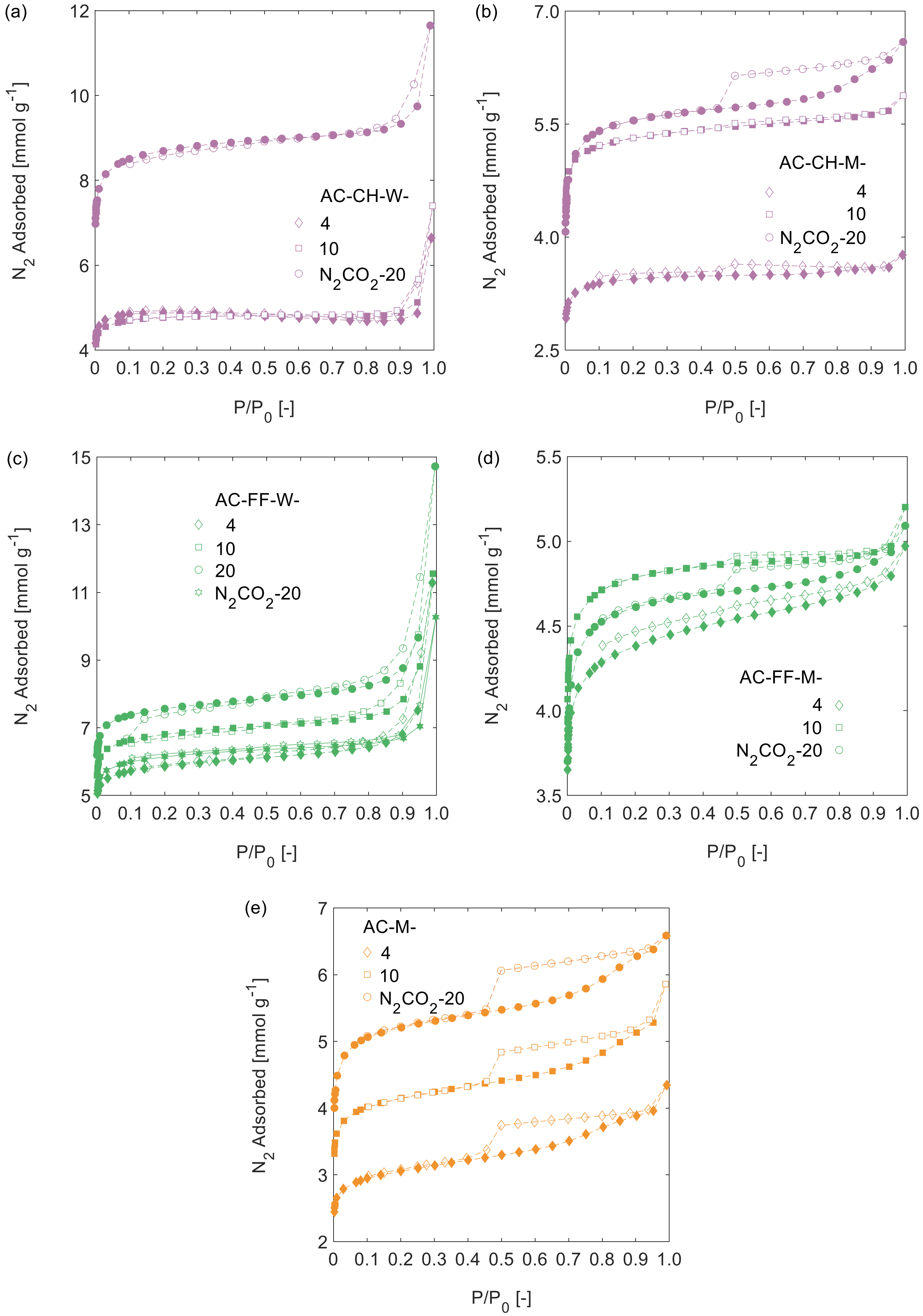


**Figure S7**. $N_2$ sorption isotherms for the activated carbons (ACs), measured at –196 °C. The starting solid (flax fiber, corn husk, or neither) and liquid (water or milk) were heated in a sealed autoclave at 220 °C to form a hydrochar that was activated in $CO_2$ at 800 °C for the indicated time. Filled symbols indicate adsorption points, and open symbols represent desorption points.

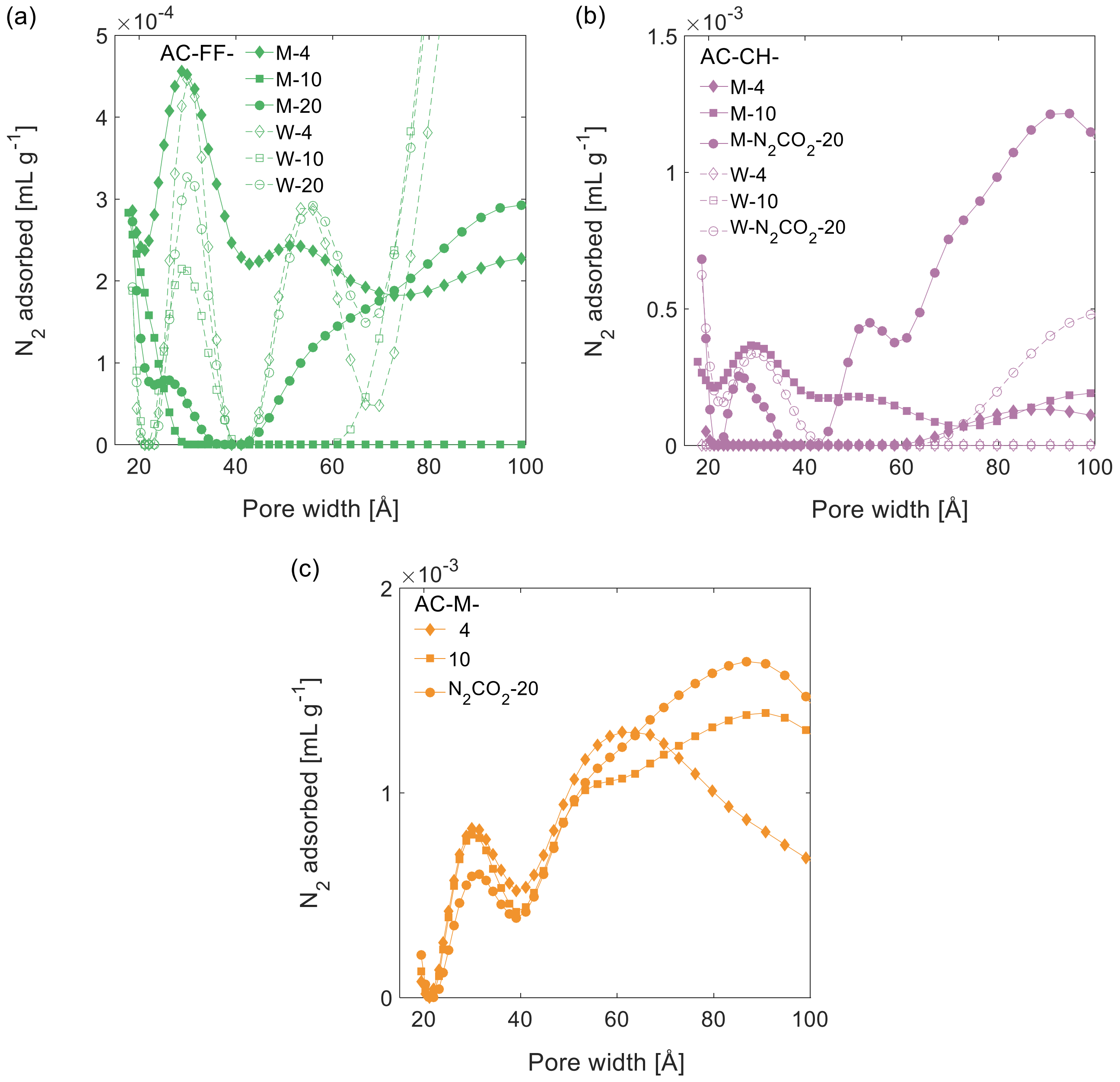


**Figure S8**. Pore size distributions derived from $N_2$ adsorption-desorption isotherms for the activated carbons (ACs). The starting solid (flax fiber, corn husk, or neither) and liquid (water or milk) were heated in a sealed autoclave at 220 °C to form a hydrochar that was activated in $CO_2$ at 800 °C for the indicated time. Filled symbols indicate adsorption points, and open symbols represent desorption points. (a) AC-FF-*y*-*t*; (b) AC-CH-*y*-*t*; (c) AC-M-*t*.

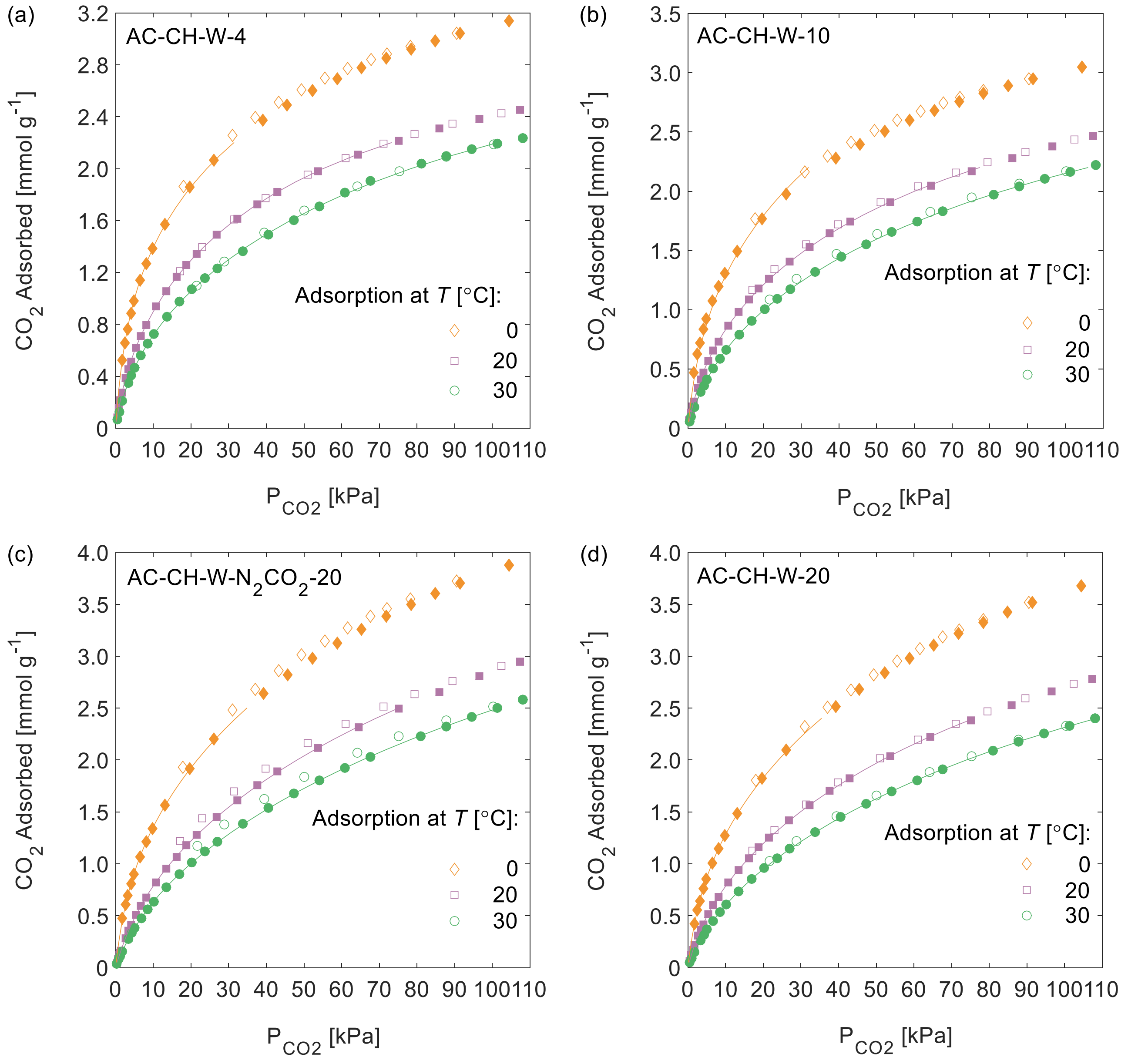


**Figure S9**. $CO_2$ sorption isotherms for the activated carbons (ACs) derived from corn husks, measured at 0, 20, and 30 °C. Corn husk and water were heated in a sealed autoclave at 220 °C to form a hydrochar, HC-CH-W, that was activated in $CO_2$ at 800 °C for the indicated time. AC-CH-W-*t* were heated to 800 ºC in $CO_2$ and held at that temperature for *t* h, whereas AC-CH-W-$N_2CO_2$-20 was heated to 800 °C under $N_2$ and then held at that temperature under $CO_2$ for 20 h. Filled symbols indicate adsorption points, and open symbols represent desorption points. Curves show the two-site Langmuir model used to calculate $Q_{st}$.

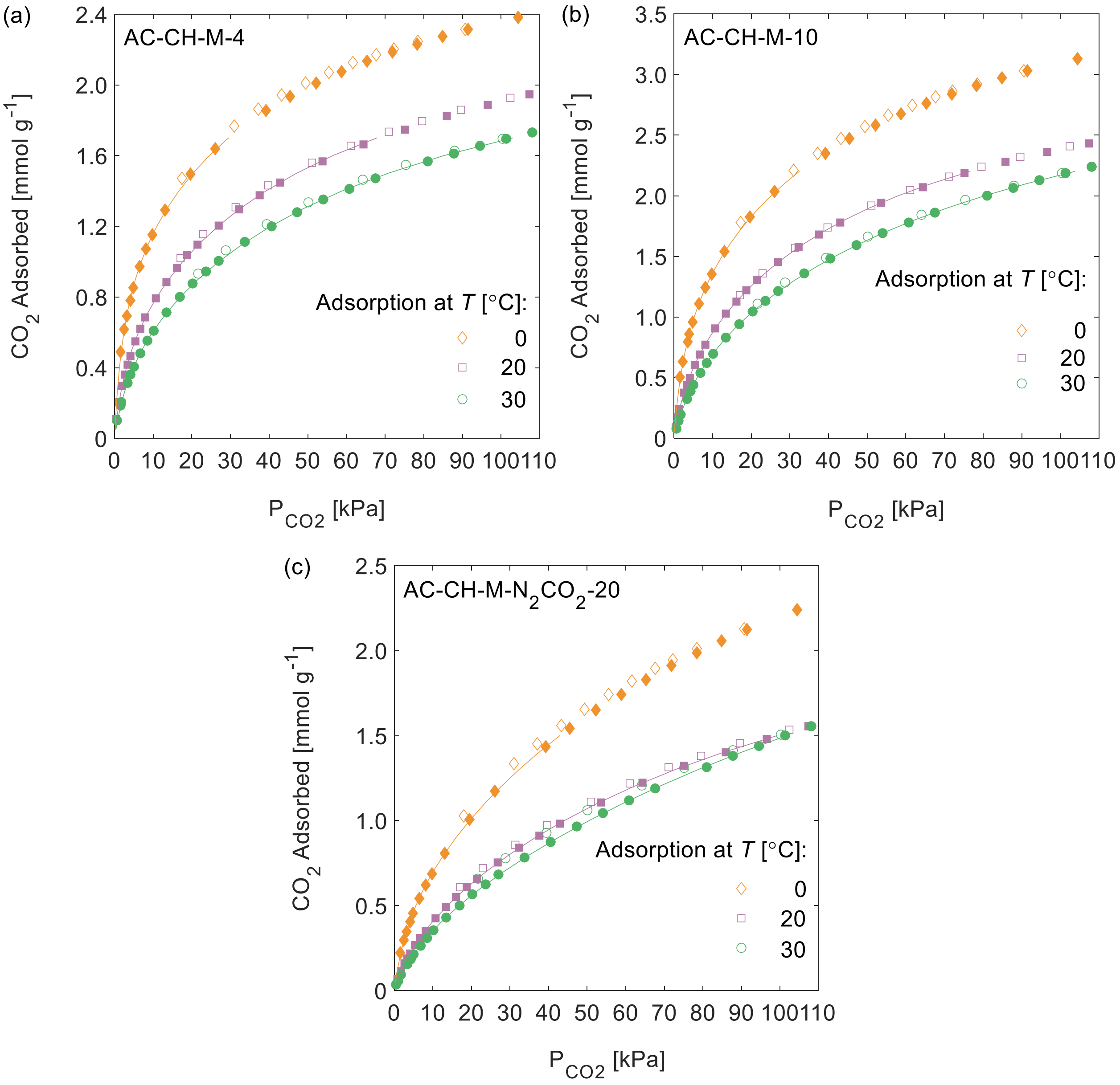


**Figure S10**. $CO_2$ sorption isotherms for activated carbons (ACs) derived from corn husks and milk, measured at 0, 20, and 30 °C. Corn husk and milk were heated in a sealed autoclave at 220 °C to form a hydrochar, HC-CH-M, that was activated in $CO_2$ at 800 °C. AC-CH-M-*t* were heated to 800 ºC in $CO_2$ and held at that temperature for *t* h, whereas AC-CH-M-$N_2CO_2$-20 was heated to 800 °C under $N_2$ and then held at that temperature under $CO_2$ for 20 h. Filled symbols indicate adsorption points, and open symbols represent desorption points. Curves show the two-site Langmuir model used to calculate $Q_{st}$.

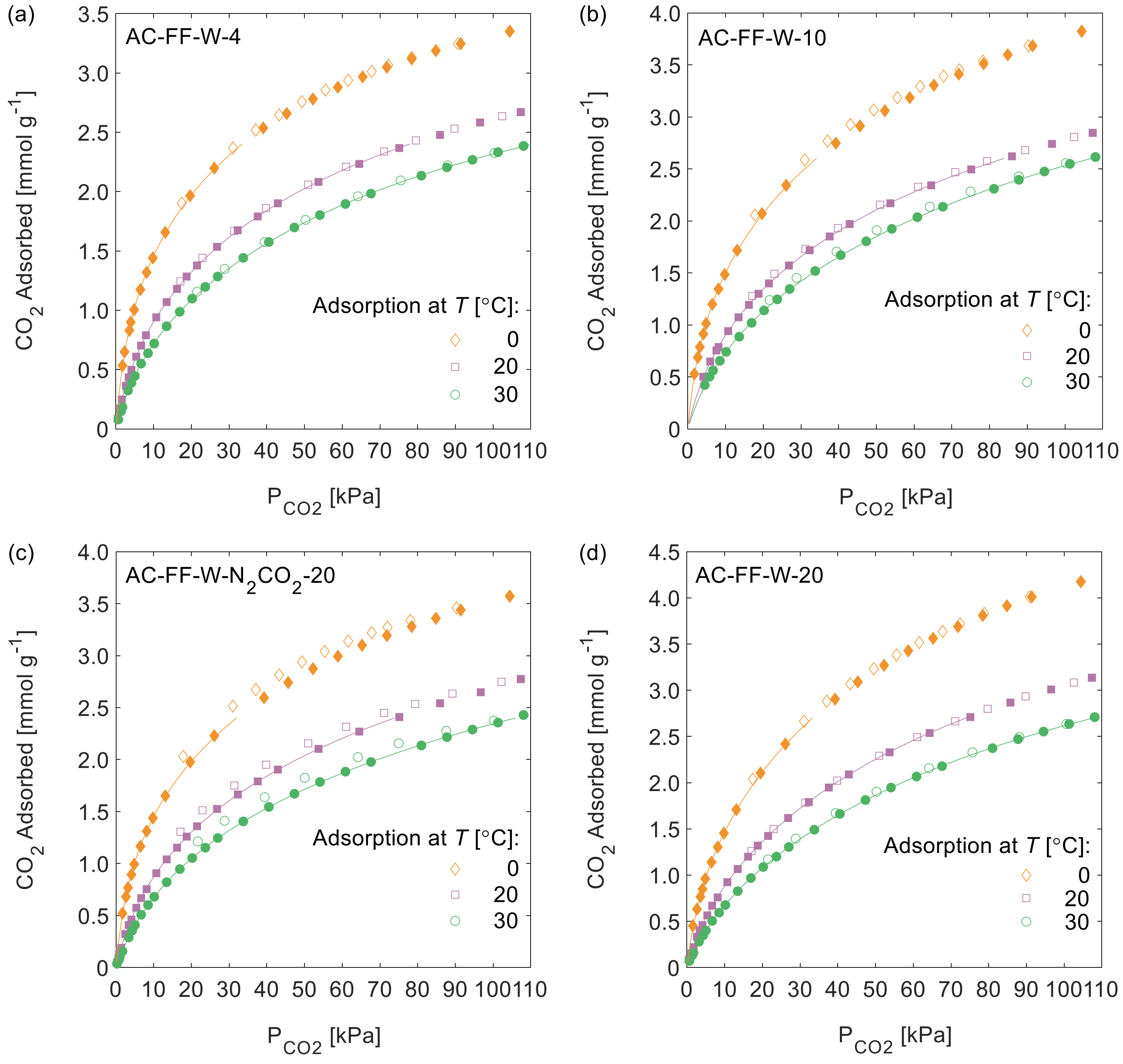


**Figure S11.** $CO_2$ sorption isotherms for the activated carbons (ACs) derived from flax fiber, measured at 0, 20, and 30 °C. Flax fiber and water were heated in a sealed autoclave at 220 °C to form a hydrochar, HC-FF-W, that was activated in $CO_2$ at 800 °C for the indicated time. AC-FF-W-*t* were heated to 800 ºC in $CO_2$ and held at that temperature for *t* h, whereas AC-FF-W-$N_2CO_2$-20 was heated to 800 °C under $N_2$ and then held at that temperature under $CO_2$ for 20 h. Filled symbols indicate adsorption points, and open symbols represent desorption points. Curves show the two-site Langmuir model used to calculate $Q_{st}$.

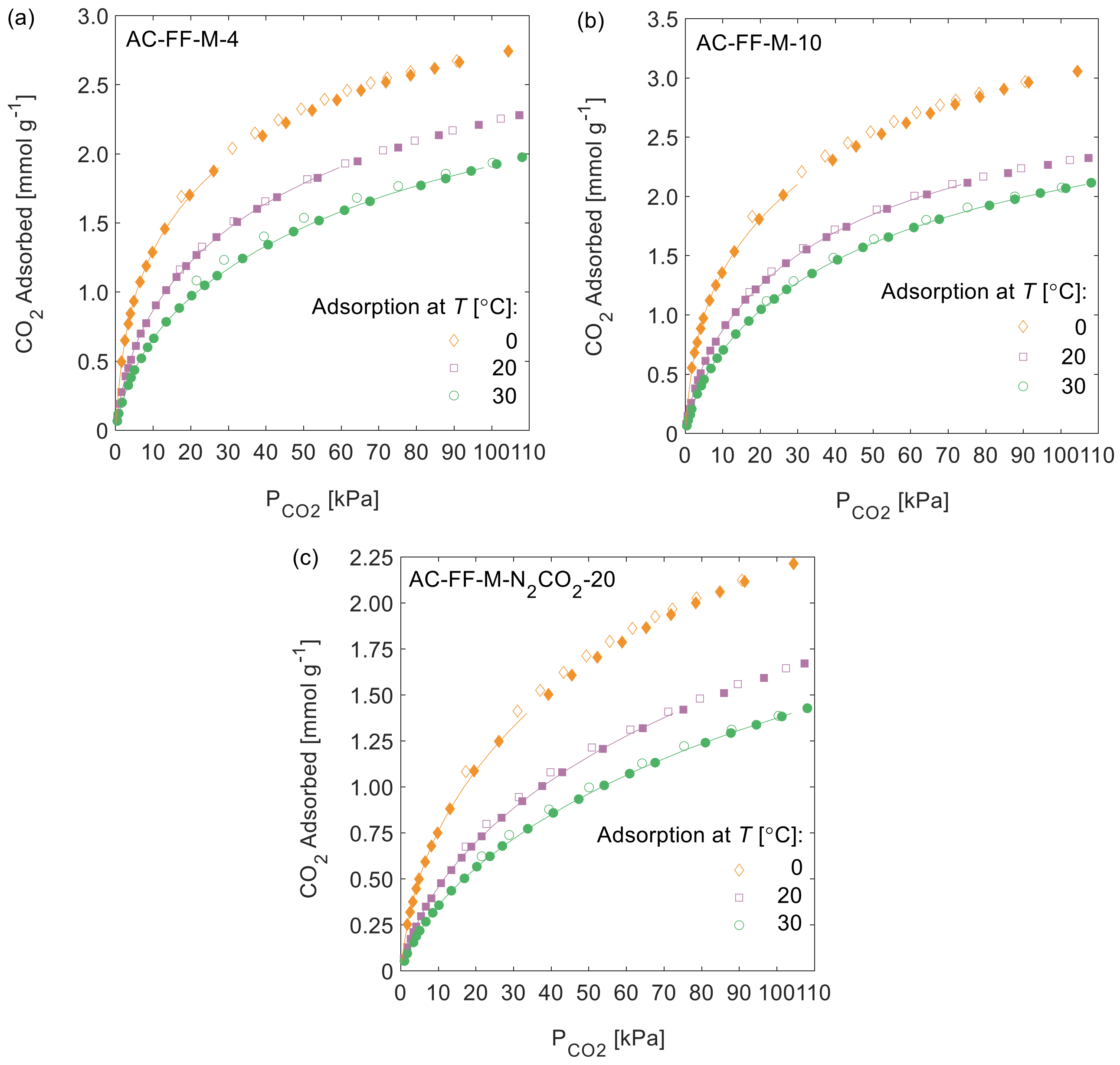


**Figure S12**. $CO_2$ sorption isotherms for the activated carbons (ACs) derived from flax fiber and milk, measured at 0, 20, and 30 °C. The flax fiber and milk were heated in a sealed autoclave at 220 °C to form a hydrochar, HC-FF-M, that was activated in $CO_2$ at 800 °C for the indicated time. AC-FF-M-*t* were heated to 800 ºC in $CO_2$ and held at that temperature for *t* h, whereas AC-FF-M-$N_2CO_2$-20 was heated to 800 °C under $N_2$ and then held at that temperature under $CO_2$ for 20 h. Filled symbols indicate adsorption points, and open symbols represent desorption points. Curves show the two-site Langmuir model used to calculate $Q_{st}$.

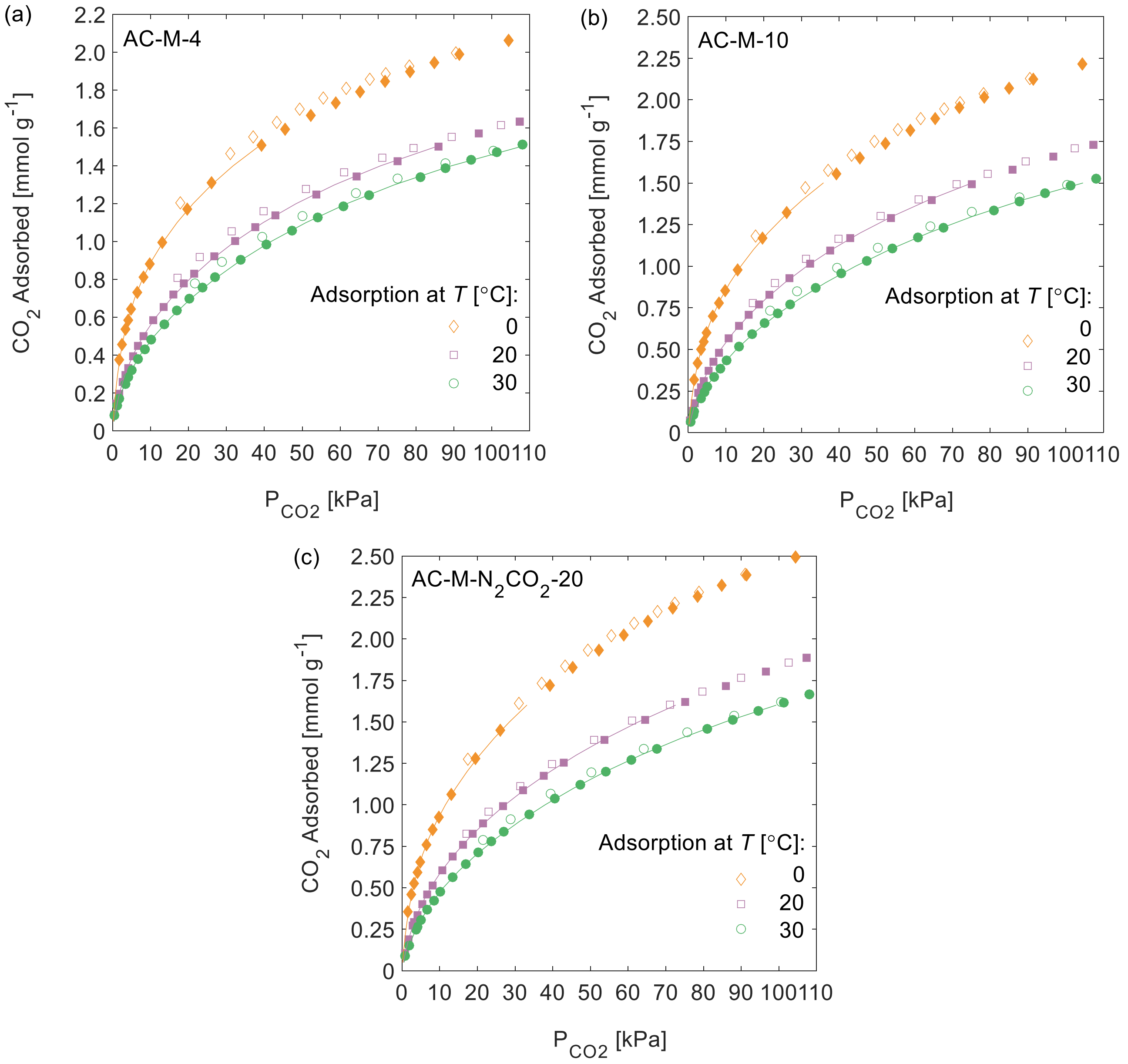


**Figure S13**. $CO_2$ sorption isotherms for the activated carbons (ACs) derived from milk, measured at 0, 20, and 30 °C. Milk was heated in a sealed autoclave at 220 °C to form a hydrochar, HC-M, that was activated in $CO_2$ at 800 °C for the indicated time. AC-M-*t* were heated to 800 ºC in $CO_2$ and held at that temperature for *t* h, whereas AC-M-$N_2CO_2$-20 was heated to 800 °C under $N_2$ and then held at that temperature under $CO_2$ for 20 h. Filled symbols indicate adsorption points, and open symbols represent desorption points.

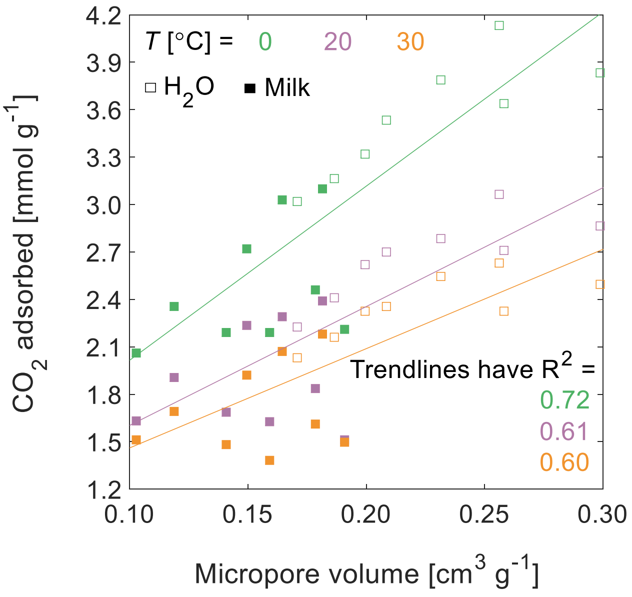


**Figure S14**. $CO_2$ adsorption under 101 kPa $CO_2$ as a function of micropore volume for activated carbons derived from hydrochars produced in water or milk. Temperatures indicate the temperature at which $CO_2$ uptake was measured, and $H_2O$/milk refers to the liquid phase used in hydrothermal carbonization.

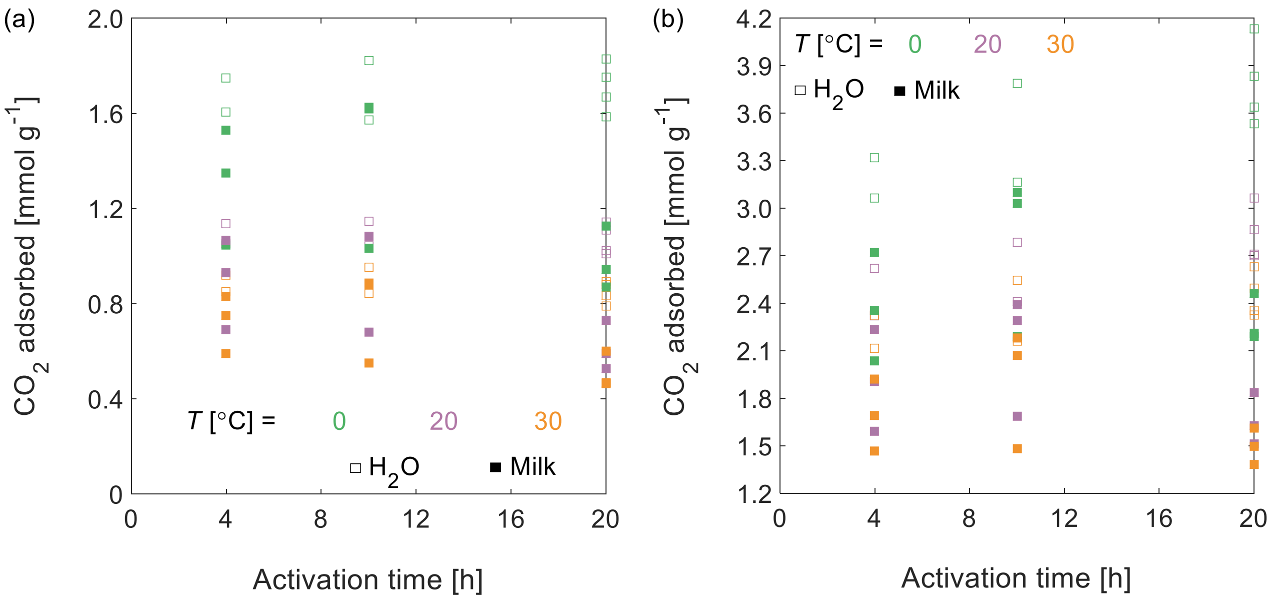


**Figure S15**. $CO_2$ uptake at (a) 15 and (b) 101 kPa $CO_2$ and 0–30 °C as a function of activation time for activated carbons derived from hydrochars produced in water and milk. Temperatures indicate the temperature at which $CO_2$ uptake was measured, and $H_2O$/milk refers to the liquid phase used in hydrothermal carbonization.

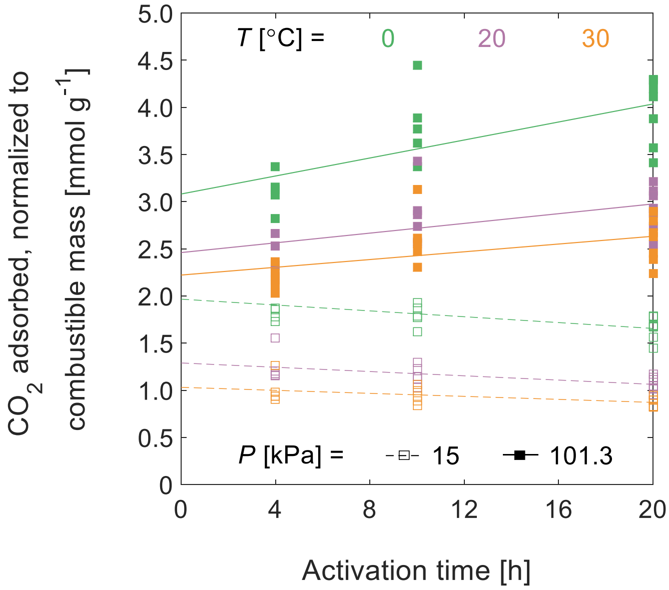


**Figure S16**. $CO_2$ uptake at 15 and 101 kPa and 0–30 °C, normalized to the mass of the solid that could be combusted by heating to 800 °C in air, as a function of activation time for activated carbons derived from hydrochars produced in water or milk.

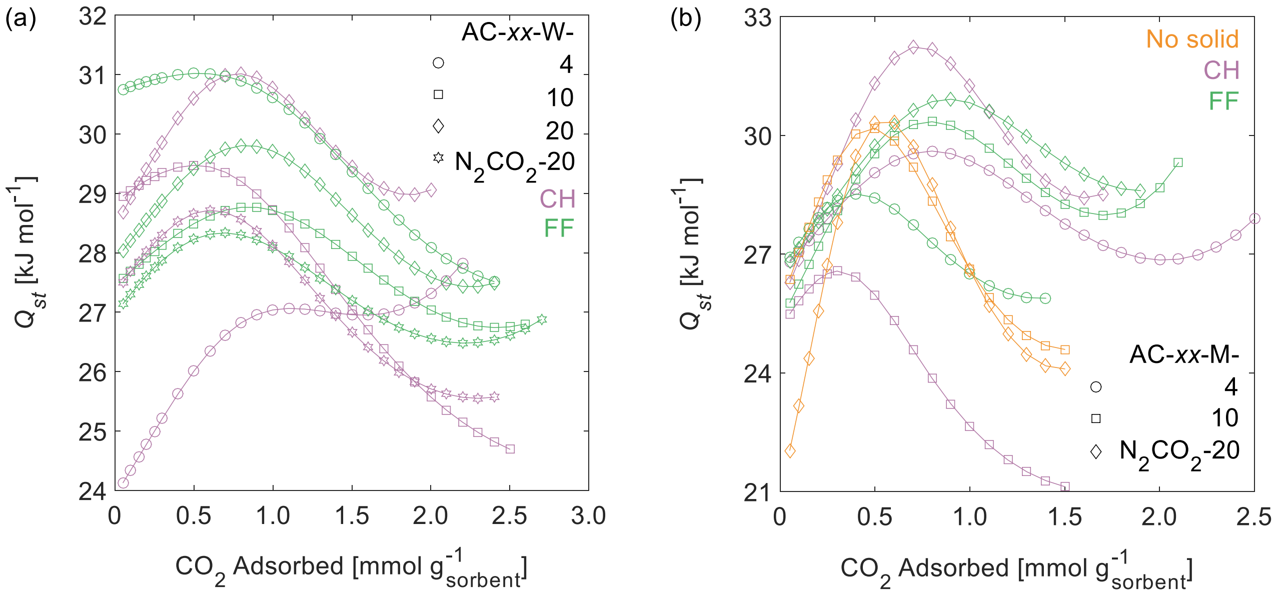


**Figure S17**. Heat of $CO_2$ adsorption, $Q_{st}$, as a function of $CO_2$ loading on activated carbons derived from hydrochars produced from corn husk, flax fiber, or neither, in (a) water or (b) milk. Activated carbons were generated by heating the hydrochar at 800 °C in $CO_2$ for the indicated time. Values of $Q_{st}$ were calculated from $CO_2$ sorption isotherms measured at 0, 20, and 30 °C; see Section S1.4 for details.

## S4. References


1. Brunauer, S.; Emmett, P. H.; Teller, E. Adsorption of Gases in Multimolecular Layers. *J. Am. Chem. Soc.* **1938**, *60*, 309-319.

2. Dubinin, M. M. Fundamentals of the theory of adsorption in micropores of carbon adsorbents: Characteristics of their adsorption properties and microporous structures. *Carbon* **1989**, *27*, 457-467.

3. Dubinin, M. M.; Zaverina, E. D.; Radushkevich, L. V. Sorption and structure of active carbons. I. Adsorption of organic vapors. *Zh. Fiz. Khim.* **1947**, *21*, 1351-62.

4. Langmuir, I. The Adsorption of Gases on Plane Surfaces of Glass, Mica and Platinum. *J. Am. Chem. Soc.* **1918**, *40*, 1361-1403.
